# circEPS15 Overexpression in Hepatocellular Carcinoma Modulates Tumor Invasion and Migration

**DOI:** 10.3389/fgene.2022.804848

**Published:** 2022-02-08

**Authors:** Bin Jiang, Maolin Tian, Gang Li, Abuduhaibaier Sadula, Dianrong Xiu, Chunhui Yuan, Yuntao Bing

**Affiliations:** Department of General Surgery, Peking University Third Hospital, Beijing, China

**Keywords:** circular RNA, hepatocellular carcinoma, eps15, microarray, ceRNA

## Abstract

Hepatocellular carcinoma (HCC) is one of the leading causes of cancer-related deaths worldwide. Recent evidence has shown that circular RNAs (circRNAs) play important roles in tissue development, gene transcription, signal regulation and tumorigenesis. However, whether circRNAs are involved in HCC progression and encode functional proteins remains largely unknown. In the present study, we aimed to explore the function and molecular mechanism of circRNAs in HCC. First, many circRNAs were found to be differentially expressed in HCC samples and paired adjacent normal liver tissues. The validation of dysregulated circRNAs by qRT-PCR revealed that circEPS15 expression was downregulated in HCC tissues, and the survival curves showed that low circEPS15 levels were associated with poor overall survival in HCC patients. Then, the overexpression of circEPS15 suppressed tumor cell invasion and migration by inhibiting the TJP1/CDH2/VIM signaling pathway and retarded cell cycle progression, which was confirmed by the Transwell culture system, wound healing assays, flow cytometry and western blot assays. After that, the spanning junction open reading frame in circEPS15 driven by IRES was shown to encode a novel protein, which was verified by western blotting with full-length, mutated, and truncated sequences of circEPS15 with a FLAG tag. Moreover, ceRNA analysis and qRT-PCR results suggest a possible circRNA (circEPS15)-miRNA-mRNA network in HCC. Collectively, our study reveals that endogenous circEPS15 plays a novel role in repressing HCC through the ceRNA network and encodes a functional protein.

## Background

Hepatocellular carcinoma (HCC) is one of the most common malignant tumors, ranking fifth in the incidence of malignant tumors and third in mortality (Olofson et al., 2018). Intrahepatic metastasis and postoperative recurrence are the main causes of death in patients with HCC. Epithelial-mesenchymal transition (EMT) is the process of transforming epithelial cells into mesenchymal cells with specific physiological or pathological phenotypes (Tam and Weinberg, 2013). In recent years, EMT has been found to be closely associated with the recurrence and metastasis of HCC and has attracted increased attention. Elucidating the molecular regulatory mechanism underlying EMT and the relationship between EMT and malignant tumors would assist in the search for new molecular targets (Tam and Weinberg, 2013; Xie and Diehl, 2013).

Circular RNA (circRNA) is different from traditional linear RNA. It has a 5′- 3′ polar covalent closed loop structure and is relatively stable, abundant, and conserved in the eukaryotic transcriptome. Recent studies have shown that the expression of circRNAs is tissue- and cell-specific (Salzman et al., 2012; Jeck et al., 2013; Lu et al., 2015). Evidence shows that circRNAs are associated with the occurrence, development, prognosis, and prognosis of cancer (Li et al., 2015; Starke et al., 2015; Chen et al., 2016; Rajappa et al., 2020; Kristensen et al., 2021); however, the role and molecular mechanism of circRNAs in HCC have not been elucidated. Therefore, elucidation of the role of circRNAs in the occurrence and development of HCC and identification of an effective target for HCC diagnosis and treatment are urgently needed.

HCC is a complex molecular process driven by gene mutations accompanied by epigenetic modifications (Bing et al., 2014). At present, there is no effective diagnostic or treatment strategy; thus, identification of potential effective diagnostic markers and therapeutic targets is urgently needed. Long noncoding RNAs (lncRNAs) have been found to be scrambled in patients with HCC, possibly having potential clinical value (He et al., 2014). However, the expression and function of circRNAs in HCC have not been elucidated.

Studies have shown that circRNA imbalance is associated with several human diseases and may be associated with the onset and progression of systemic lupus erythematosus, diabetes, and Alzheimer’s disease (Zhao et al., 2016; Li C. et al., 2018; Li H. et al., 2018). Other studies have shown that circRNAs are involved in the occurrence and development of tumors, including colon, gastric, and cervical cancers (Gao et al., 2017; Hsiao et al., 2017; Huang et al., 2017; Weng et al., 2017). Although circRNAs have received increased attention, the biological functions and mechanisms of several circRNAs have yet to be elucidated, and it remains unknown whether these circRNAs are transcribed.

The molecular mechanism of circRNA biological function primarily includes miRNA absorption through the sponge effect, which affects the biological function of the miRNA (Zheng et al., 2016; Weng et al., 2017). circRNA is also a biological regulator that binds to proteins (Zeng et al., 2017) and can translate functional proteins, thereby influencing glioma tumorigenesis (Yang et al., 2018). However, whether circRNAs play a biological role in HCC and the underlying mechanism have not been elucidated.

miRNAs in circulation can be used as potential biomarkers for pancreatic cancer, Barrett’s esophagus and esophageal adenocarcinoma (Gablo et al., 2019; Lv et al., 2020). However, although many clinical studies have been conducted on HBV-related HCC and hepatitis C virus (HCV)-related HCC (Li et al., 2019; Morishita et al., 2020), the instability of miRNAs and difficulty in reproducing results under different experimental conditions make miRNAs poor biomarkers for clinical practice. In contrast, circRNA is more stable due to its closed-loop structure and tolerance to several RNA enzymes present in the environment. Moreover, due to its tissue-specific expression (Melo et al., 2015), circRNA can be an ideal biomarker for predicting the occurrence, progression, and prognosis of disease.

Further investigations into the mechanism of circRNA may elucidate its potential therapeutic use in the future. Additionally, due to its stability, circRNA can be used as a continuous cell regulator. For example, a specific circRNA can be synthesized through genetic engineering to play a specific biological role by binding to a specific miRNA, competing for binding to ribosomal proteins, or translating a small polypeptide. One study showed that an overexpressed synthesized circRNA could absorb miRNA-122 through sponge-like interactions, thereby affecting the HCV replication cycle by inhibiting its protein synthesis (Jost et al., 2018). Therefore, synthetic circRNAs have broad applications in molecular medicine.

In this study, we investigated the cancer inhibitory role of circRNA-100226 in HCC using paired human HCC and paracarcinoma tissues. Using the human reference genome, we further estimated that the circRNA located at chr1:51868106–51874004 is derived from EPS15 (epidermal growth factor receptor pathway substrate 15), which is located on chromosome 1p32.3. Thus, we referred to circRNA-100226 as “***circESP15***”. We also overexpressed circEPS15 to further investigate the mechanisms of tumor growth.

## Materials and Methods

### Ethics Statement

All clinical and experimental procedures were reviewed and approved by the Ethics Committee of Peking University Third Hospital. Written consent was obtained from all patients prior to surgery. All protocols were performed in accordance with the Declaration of Helsinki (General Assembly of the World Medical Association, 2014).

### Patients and Specimens

A total of 71 HCC tissues and matched adjacent nontumor tissues were obtained from patients who underwent surgery in the Third Hospital, Peking University. None of the patients had received chemotherapy, radiotherapy, or any other anticancer therapy before surgery. The diagnoses were histologically confirmed by experienced pathologists. Paired tissue specimens (tumor and adjacent normal tissues) were collected from the patients, immediately frozen in liquid nitrogen, and stored at −80°C until use.

### CircRNA Microarray Hybridization and Data Analysis

Total RNA was digested with RNase R (20 U/μL, Epicentre, Madison, WI, United States) to remove linear RNAs and enrich circular RNAs. The enriched circular RNAs were amplified and transcribed into fluorescent cRNA utilizing a random priming method (Super RNA Labeling Kit; Arraystar, Rockville, MD, United States). The labeled cRNAs were hybridized onto the Human circRNA Array (8 × 15K, Arraystar). The slides were incubated for 17 h at 65°C in a hybridization oven (Agilent Technologies, Santa Clara, CA, United States). CircRNAs with significant differential expression between the HCC and paired normal tissues (fold change (FC) ≥ 2 and *p* ≤ 0.05) were identified by volcano plot filtering. Hierarchical clustering was performed to determine the distinguishable expression pattern of circRNAs among the samples. The raw microarray data were uploaded to NCBI, and the GEO access number is GSE164803.

### GO and KEGG Pathway Analysis

GO and KEGG pathway analyses were performed using standard techniques. GO analysis was performed to explore the functional roles of DE mRNAs and miRNAs in terms of “biological processes (BPs),” “cellular components (CCs)” and “molecular functions (MFs)” (http://www.geneontology.org). Pathway analysis was performed using the KEGG (http://www.genome.jp/kegg) database.

### Prediction of Target miRNAs and Target mRNAs for circEPS15 and Biological Pathway Enrichment Analysis

CircRNAs act as miRNA sponges by binding miRNA response elements (MREs) in a competitive manner, preventing the inhibitory effects of miRNAs on target genes and increasing target gene amounts (Salmena et al., 2011; Phelps et al., 2016). To identify potential targets of miRNAs, we predicted the target/miRNAs with homemade miRNA target prediction software based on TargetScan and miRanda (Garcia et al., 2011; Li et al., 2014). First, the circRNA/miRNA interaction was predicted. Next, databases were used to identify miRNA-mRNA pairs. Finally, circRNA-miRNA-mRNA networks were constructed. GO and KEGG analyses were performed for targeted mRNAs.

### Cell Culture

Human HCC cell lines (BEL-7402, BEL-7404, Huh7, Hep3B, and HepG2) and the normal hepatocyte cell line HL-7702 were purchased from the Shanghai Institute of Cell Biology (Shanghai, China) with a certificate of authenticity for each cell line. The samples were maintained in DMEM/RPMI 1640 (Gibco BRL, Grand Island, NY, United States) with 10% heat-inactivated fetal calf serum, 2 mM l-glutamine, and 100 U/mL penicillin–streptomycin mixture (Gibco BRL, Grand Island, NY, United States) at 37°C in 5% CO_2_. After the cells were cultured for several months, all cell lines were evaluated for genetic identification prior to the experiments. Based on the appraisal reports provided by CoBioer Biosciences Co., Ltd. (Nanjing, China), no mutations or contaminations were found in our cell lines.

### Quantitative Reverse Transcription Polymerase Chain Reaction

The cDNAs for circRNA, mRNA, or miRNA measurement were synthesized using random, oligo (dT)18, or stem-loop primers, respectively, using a RevertAid First Strand cDNA Synthesis Kit (Thermo Fisher Scientific). qRT-PCR was performed in triplicate using Maxima SYBR Green qPCR Master Mix (Thermo Fisher Scientific) on the CFX connect real-time system (Bio-Rad, Hercules, CA, USA). All primers used for qRT-PCR were designed using Primer 5.0 software (Premier, Canada) and synthesized by TsingKe Biotech (Chengdu, China). The primer sequences for the related qPCR validation are shown in Supplementary Table S2.

### circEPS15-Overexpressing Vector Construction and Transduction

The circEPS15-overexpressing plasmid was synthesized and cloned into the adenoviral vector GV486 (GeneChem, Shanghai, China) with a Flag tag. Full-length circEPS15 was serially diluted with X-tremeGENE siRNA transfection reagent (Roche, Mannheim, Germany). For western blot analysis, cell proteins were prepared 72 h post-transfection.

### Wound Healing Assay

The cells were seeded in 6-well plates and transfected following the manufacturer’s instructions. When the cell confluence reached approximately 80% at 48 h post-transfection, the cells were serum-starved for 12 h and scratched using the fine end of a 100-µL pipette tip. Wound healing was observed at different time points within the scratch line. Every 24 h, scratch lines were imaged using an inverted Leica phase-contrast microscope (DFC 300 FX, Wetzlar, Germany) under a 20× objective lens. Image processing and analysis in Java was performed to obtain the wound-healing assay results. Duplicate wells were examined for each condition, and each experiment was repeated three times.

### Transwell Assay

A cell invasion assay was performed in a Transwell chamber (24-well type, 8 mm pore size, Corning, Inc., Corning, NY, United States). BD Matrigel Basement Membrane Matrix (BD Biosciences, Franklin Lakes, NJ, United States) was used according to the manufacturer’s instructions. Next, 0.5 ml of serum-free medium was added to the upper chambers; DMEM containing 10% FBS was added to the bottom chambers. Equal numbers (1 × 10^5^) of cells were plated in the upper chambers of the quadruplicate wells and incubated at 37°C for 72 h. The cells were then fixed with paraformaldehyde and stained with crystal violet to visualize the nuclei. Average values were calculated from the results of three independent experiments. Image processing and analysis in Java was performed to obtain the Transwell assay results.

### Cell Cycle and Apoptosis Assay

For analysis of the cell cycle and apoptosis, 3 × 10^5^ treated cells were seeded in 6-well plates and cultured for 48 h at 37°C. For cell cycle analysis, the cells were digested using trypsin (HyClone, Logan, UT, United States), washed twice with phosphate-buffered saline (PBS), and fixed in 70% ethanol overnight at 4°C. The cells were centrifuged at 500 ×*g* for 5 min, washed twice with cold PBS, and centrifuged. After the cells were treated with RNase A (0.1 mg/ml) and propidium iodide (PI, 0.05 mg/ml, 4A Biotech, Beijing, China) for 30 min at 37°C, cell cycle analysis was performed using fluorescence-activated cell sorting flow cytometry (Beckman Coulter, Palo Alto, CA, USA). For analysis of apoptosis, the cells were trypsinized followed by two PBS washes. The cells were stained using the Annexin V/PI detection kit (4A Biotech) for 5 min at 25°C. Apoptotic cells were measured using flow cytometry (Beckman Coulter). All experiments were repeated at least three times.

### Western Blot Analysis

Exosomes were lysed with RIPA Lysis Buffer I (Sangon Biotech, Cat: C500005) to obtain the total protein. The protein concentration of each sample was determined using a NanoDrop™ spectrophotometer (Thermo Fisher Scientific, Cat: A30221). Protein (100 µg) from each sample was subjected to SDS-PAGE (4% stacking and 10% separating gels) and then transferred overnight onto polyvinylidene fluoride membranes (Millipore, Billerica, MA, United States). The primary antibodies used here included anti-human TJP1 antibody (Abcam, Cat: AB216880), anti-CDH2 antibody (Abcam, Cat: AB76057), anti-VIM antibody (Abcam, Cat: AB 137321), anti-EPS15 antibody (Abcam, Cat: AB224811), anti-GAPDH antibody (Abcam, Cat: AB8245), and anti-Flag antibody (Abcam, Cat: AB1162).

### IRES Prediction

IRESfinder (Zhao et al., 2018) was used to identify the IRES sequence of circRNAs. Each sequence of circRNA was evaluated by the sliding window approach, with a window size of 174 nt and a step size of 50 nt. The region with the highest score was considered the IRES sequence of circRNA.

### Statistical Analyses

All statistical analyses were performed using SPSS version 21.0 (SPSS, Inc., Chicago, IL, United States) and Prism version 5.0 (GraphPad Software, La Jolla, CA, United States) software. Categorical variables are expressed as a count or percentage and were tested using χ2 or Fisher’s exact test, as deemed appropriate. Continuous data are shown as the mean ± standard deviation (SD) and were compared using Student’s t test, one-way analysis of variance, or Mann-Whitney test as deemed appropriate. Correlations were calculated using Pearson’s correlation analysis. The median expression of target genes was used as the cutoff value to stratify patients into high and low expression groups. Survival curves were plotted using the Kaplan-Meier method and compared using the log-rank test. All tests were 2-sided, and results with *p* < 0.05 were considered statistically significant.

## Results

### circRNA Expression Profiles in HCC

**FIGURE 1:**
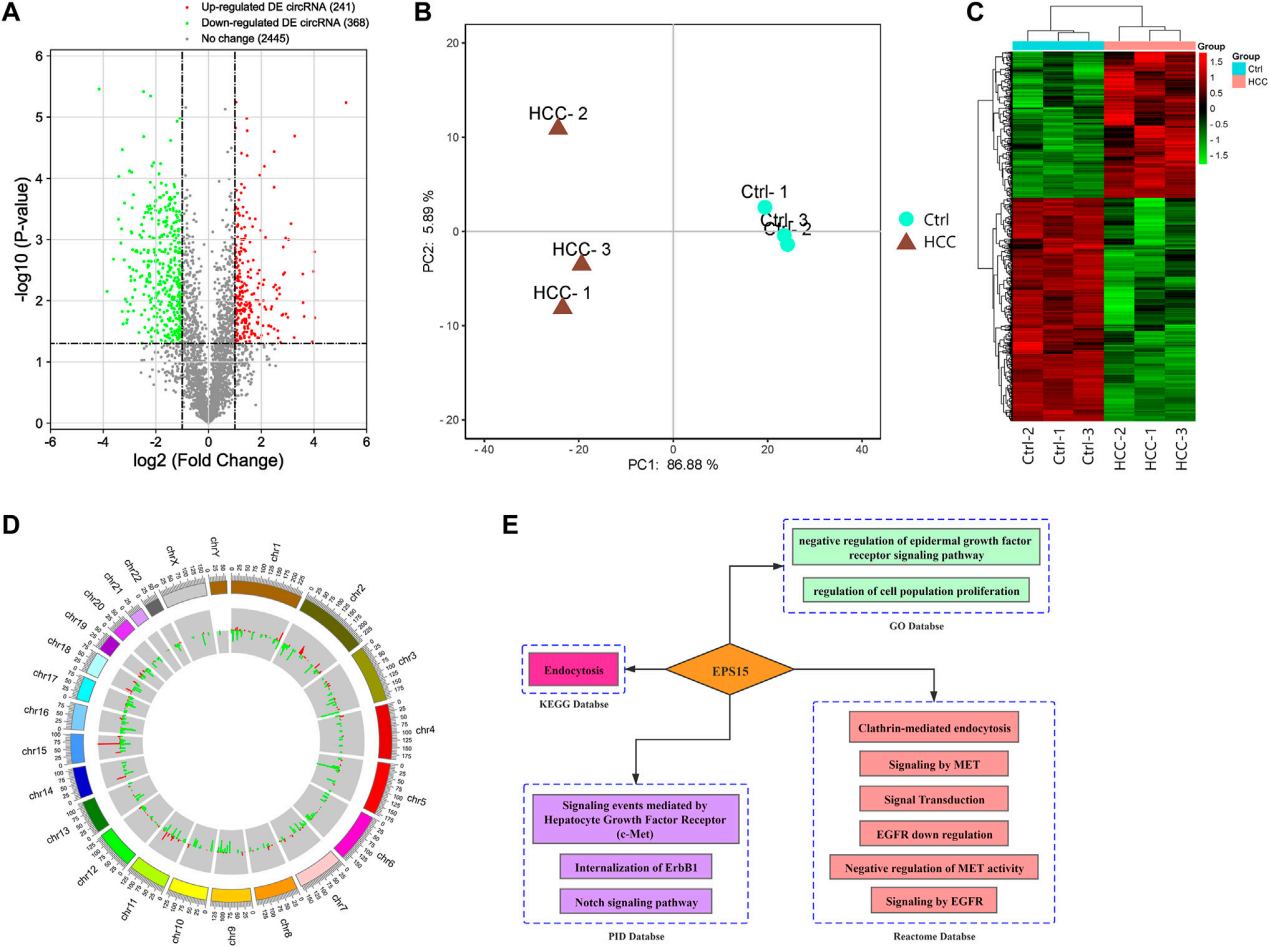
**(A)** Volcano plot of differential expression of circRNAs between the HCC tissues and the control tissues. The *X*-axis and *Y*-axis of the volcano plot represent the log of the fold change (FC) so that changes in both directions (up and down) appear equidistant from the center and a negative log of the *p* value. **(B)** Principal component analysis (PCA) indicated that there was a distinct classification between the HCC group and the control group, and the biological repeatability of samples in the group was relatively good. **(C)** Heatmap of differentially expressed (DE) circRNAs: the DE circRNAs in the HCC tissues compared with the control tissues (*n* = 3). The FC cutoff was 2. **(D)** Circle plot of DE circRNA distribution in different chromosomes. **(E)** Biological function analysis of EPS15. GO database, KEGG database PID database and Reactome database enrichment analysis indicated that the host gene EPS15 was involved in the MET pathway.

A microarray was performed to assess differentially expressed circular RNAs in the HCC vs. control groups (*n* = 3). circRNAs were considered differentially expressed with FC > 2 and *p* value <0.05. Six hundred and nine differentially expressed circRNAs were identified: 241 with upregulated and 368 with downregulated expression (Figure 1A). Principal component analysis was performed comparing the HCC and ctrl groups. Biological repeatability among the samples was relatively good (Figure 1B), as revealed by cluster analysis (Figure 1C). The chromosomal distribution of differentially expressed circRNAs is shown in Figure 1D. Raw intensity was set to >1,000 in each of the HCC and control groups, and the differentially expressed circRNAs up- and downregulated expression with a relatively high abundance and the top 10 multiples of differences were selected, as shown in Supplementary Table S1.

With the KOBAS V3.0 database (http://kobas.cbi.pku.edu.cn/), host circRNA genes with raw intensity >1,000 and with the top 10-fold up- and downregulation scores were annotated. We found that circRNA-100226, corresponding to endothelial growth factor receptor pathway substrate 15, is involved in the MET pathway and has related regulatory functions, as shown in Figure 1E. Based on the finding that the host gene EPS15 is involved in the MET pathway, we chose circEPS15 (circRNA-100226) as a candidate molecule to verify the related functional mechanisms.

### qRT-PCR Validation of circEPS15

**FIGURE 2:**
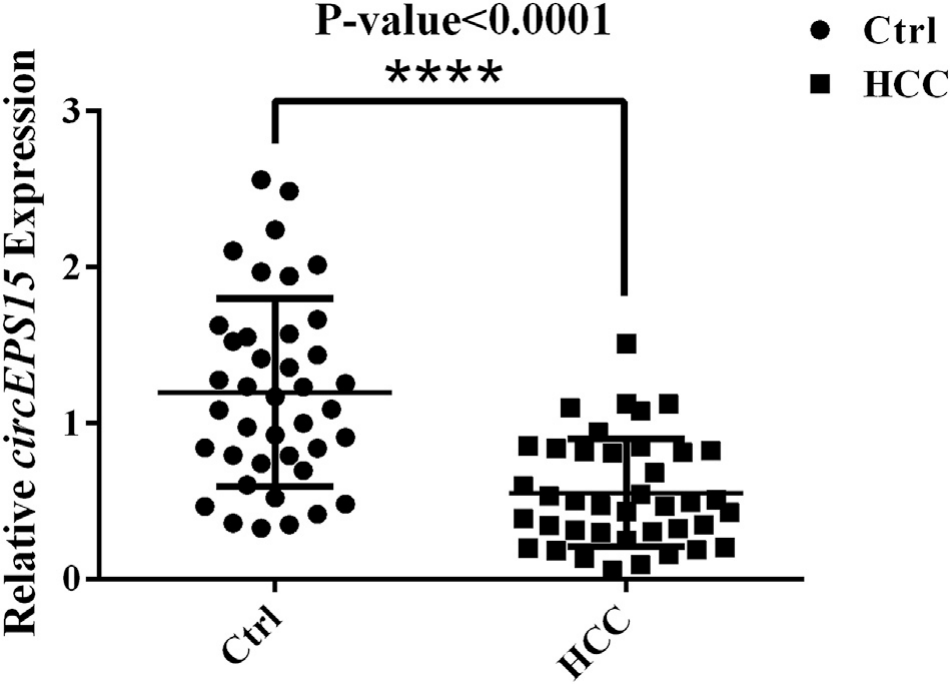
Expression levels of circEPS15 in 40 randomly selected paired hepatoma and adjacent tissue samples detected with junction primers (*p* < 0.01).

To verify the expression of circEPS15, we selected 40 random hepatoma samples and their adjacent tissues for detection by qRT-PCR with splice junction-specific primers. The primers are listed in Supplementary Table S2. circEPS15 had downregulated expression in HCC tissues, which was consistent with our microarray analysis (Figure 2).

### Prognostic and Diagnostic Value of circEPS15

**FIGURE 3:**
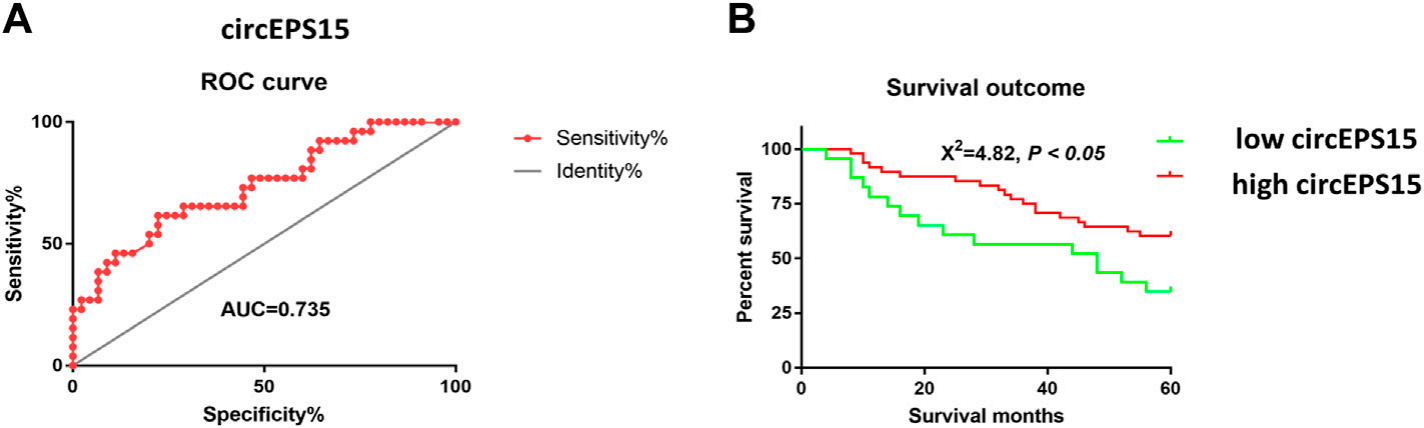
**(A)** Combined with the prognostic results from these patients, an ROC curve was constructed, and an AUC of 0.735 for circEPS15 was obtained, indicating that the expression of circEPS15 has predictive value for prognosis of HCC patients. **(B)** Kaplan–Meier survival curves depict the correlation between circEPS15 and the overall survival of HCC patients. The patients were stratified into two groups according to a cutoff value of 0.8796 for circEPS15. Patients with low circEPS15 expression had a shorter overall survival than patients with high circEPS15 expression. A total of 71 patients were included in each experiment, and values indicate the mean ± SD of three independent experiments. Student’s two-tailed paired *t* test and log-rank test were used for statistical analysis of these data.

Seventy-one patients completed the clinical follow-up and had available survival information from which receiver operating characteristic (ROC) analysis was performed to determine whether circEPS15 had diagnostic significance. The results showed that the area under the curve (AUC) of circEPS15 was 0.735, which confirmed that circEPS15 was diagnostically significant for HCC (Figure 3A). Kaplan-Meier analysis of the correlation between circEPS15 and patient survival showed that low circEPS15 levels were significantly associated with poor overall survival (Figure 3B).

### Correlation Between circEPS15 Expression and HCC Patients’ Clinical Characteristics

To further confirm the role of circEPS15 in HCC, we analyzed the relationship between circEPS15 expression in HCC tissues and the clinicopathological characteristics of HCC patients. As shown in Table 1, there was a significant correlation between the low expression level of circEPS15 in HCC tissues and tumor differentiation (*p* = 0.017) and vascular invasion (*p* = 0.011). In addition, other characteristics of HCC, including the AFP level, tumor number, tumor size and BCLC stage, were not associated with circEPS15 expression. This correlation study may imply that circEPS15 is involved in the tumorigenesis and intrahepatic metastasis of HCC.

**TABLE 1:** Correlation between *circEPS15* expression and HCC clinical characteristics.

	Total	*circEPS15*		*p*-value
		Low (≤0.8976)	High (>0.8976)	
Age				
<60	48	32	16	0.515
≥60	23	16	7	
Gender				
Male	51	33	18	0.295
Female	20	15	5	
HBV				
<10^∧^3	43	31	12	0.228
>10^∧^3	12	17	11	
AFP				
<400	17	11	6	0.494
≥400	54	37	17	
ALB				
<35 g/L	13	10	3	0.329
≥35 g/L	58	38	20	
Tbil				
<23.3	55	38	17	0.416
≥23.3	16	10	6	
PT				
<12.8	47	34	13	0.177
≥12.8	24	14	10	
Tumor diameter				
<5 cm	37	27	10	0.225
≥5 cm	34	21	13	
Tumor differentiation				
M + L	32	17	15	0.017
H + M	39	31	18	
Tumor number				
1	57	37	20	0.260
2	14	11	3	
Intravascular tumor thrombus				
No	28	14	14	0.011
Yes	43	34	9	

### Effects of circEPS15 on HCC Tumor Cell Proliferation and Cell Cycle Progression

**FIGURE 4:**
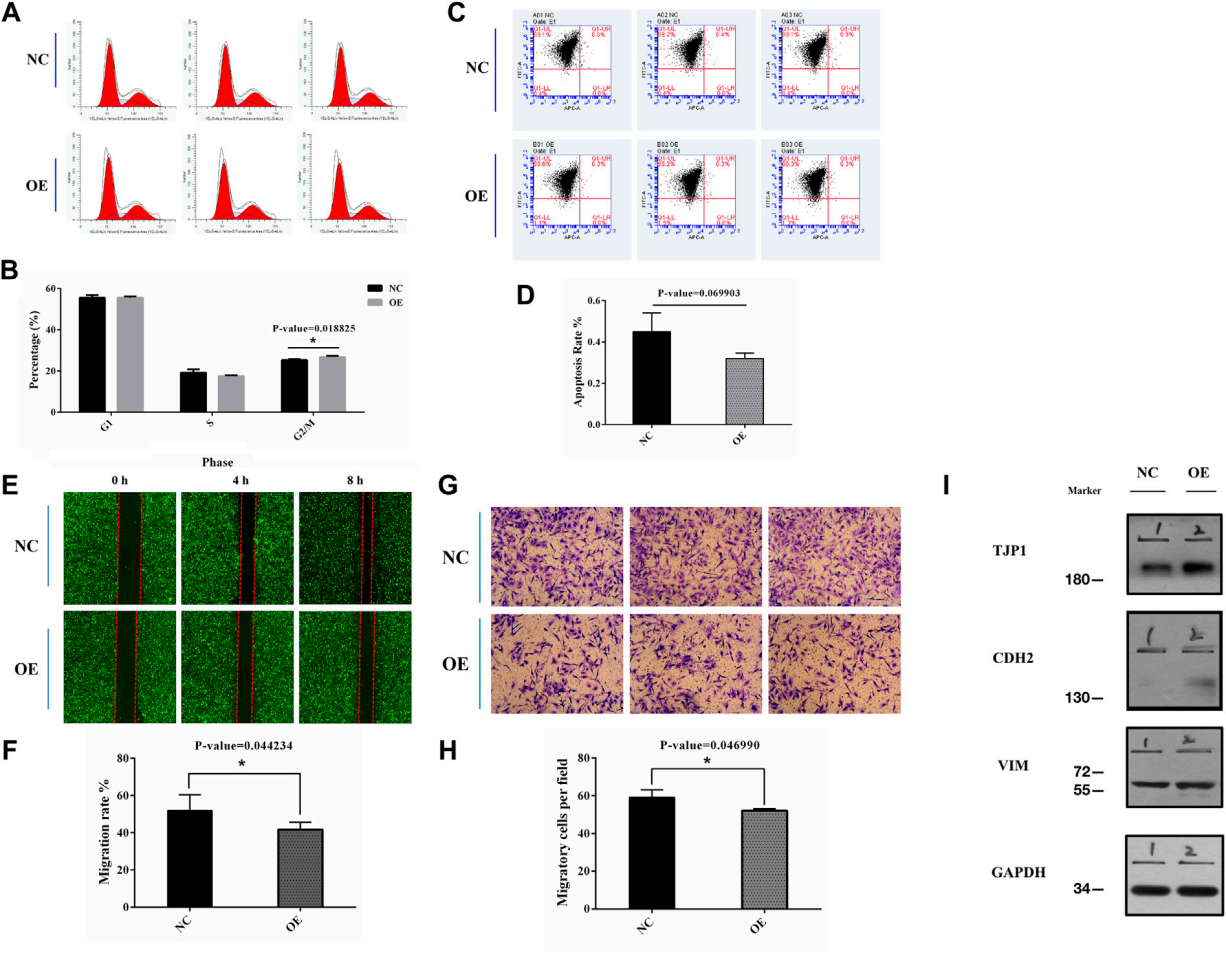
**(A,B)** Flow cytometry was used to detect the cell cycle in transfected BEL-7402 cells (*p* = 0.018825). **(C,D)** Flow cytometry was used to detect cell apoptotic progression in transfected BEL-7402 cells (*p* = 0.069903). **(E,F)** Migration of cells overexpressing circEPS15 or the negative control was measured in a wounding assay at 0 and 48 h. The bar chart illustrates the relative migratory distance of cells into the wounded region. The data are the mean ± SEM of three independent experiments (*p* = 0.044234, compared to 0 h). **(G,H)** A Transwell assay was used to evaluate the invasion of cells overexpressing circEPS15 or the empty vector control after 48 h. The bar chart illustrates the relative percentage of migrating cells. The data are the mean ± SEM of three independent experiments (*p* = 0.046990, compared to the mock control). **(I)** Representative western blotting images show that TJP1 expression in BEL-7402 cells decreased and CDH2 expression increased in circEPS15-overexpressing cells compared with those transfected with the empty vector. However, there was no significant change in VIM expression.

To investigate the specific function of circEPS15 in HCC formation and development, we confirmed that the tumor cell line BEL-7402 had low expression of circEPS15 compared to the normal cell line HL-7702 among 6 cell lines associated with HCC (Supplementary Figures S1A). We constructed an adenovirus vector to overexpress circEPS15 in the BEL-7402 cell line (Supplementary Figures S1B). Following transfection, both the fluorescence intensity of GFP and the expression of circEPS15 increased in BEL-7402 cells, indicating successful transfection and overexpression of circEPS15 in BEL-7402 cells (Supplementary Figures S1C,D); therefore, we used BEL-7402 cells for further study. We investigated the effect of circEPS15 overexpression on the cell cycle by measuring the percentage of HCC cells in different cell cycle stages (e.g., G1/G0, S, and G2/M) and found that circEPS15 overexpression increased the percentage of cells in G2/M compared to that of the vector control (Figures 4A,B). In addition, no significant difference in apoptosis was observed between the transfected cells and the negative controls (Figures 4C,D). These results indicated that circEPS15 elicited cell cycle arrest.

### circEPS15 Overexpression Suppressed HCC Cell Migration and Invasion

A correlation study might show that circEPS15 is involved in HCC tumorigenesis and intrahepatic metastasis. Wound healing and Transwell assays indicated that circEPS15 significantly decreased HCC cell migration and invasion compared to the negative control (NC) (Figures 4E–H).

Through the Gene Ontology (GO, http://geneontology.org/), Kyoto Encyclopedia of Genes and Genomes (KEGG, https://www.genome.jp/kegg/), and Reactome (https://reactome.org/) databases, we identified the TJP1, CDH2, and VIM proteins involved in epithelial cell differentiation, cell junctions, cell adhesion molecules, the Rho GTPase cycle, and other pathways (Supplementary Figures S2A). Using the HCCDB database (http://lifeome.net/database/hccdb/home.html), we found that TJP1 was highly expressed in HCC compared with adjacent tissues, and a good prognosis was found with high expression (Supplementary Figures S2B,C). CDH2 was expressed at a low level, but high expression could be observed in individual datasets, and a good prognosis was linked to low expression (Supplementary Figures S2E,D). The expression trend of VIM in HCC was unstable, and the prognosis could not be accurately determined (Supplementary Figures S2F,G). We examined the TJP1, CDH2, and VIM expression levels using western blotting to further confirm that circEPS15 affected cell migration and invasion. The results showed that the TJP1 and CDH2 expression levels increased in cells overexpressing circEPS15, while that of VIM did not change significantly (Figure 4I).

### circRNA-miRNA Interactions and Competing Endogenous RNA Regulatory Network Analysis

**FIGURE 5:**
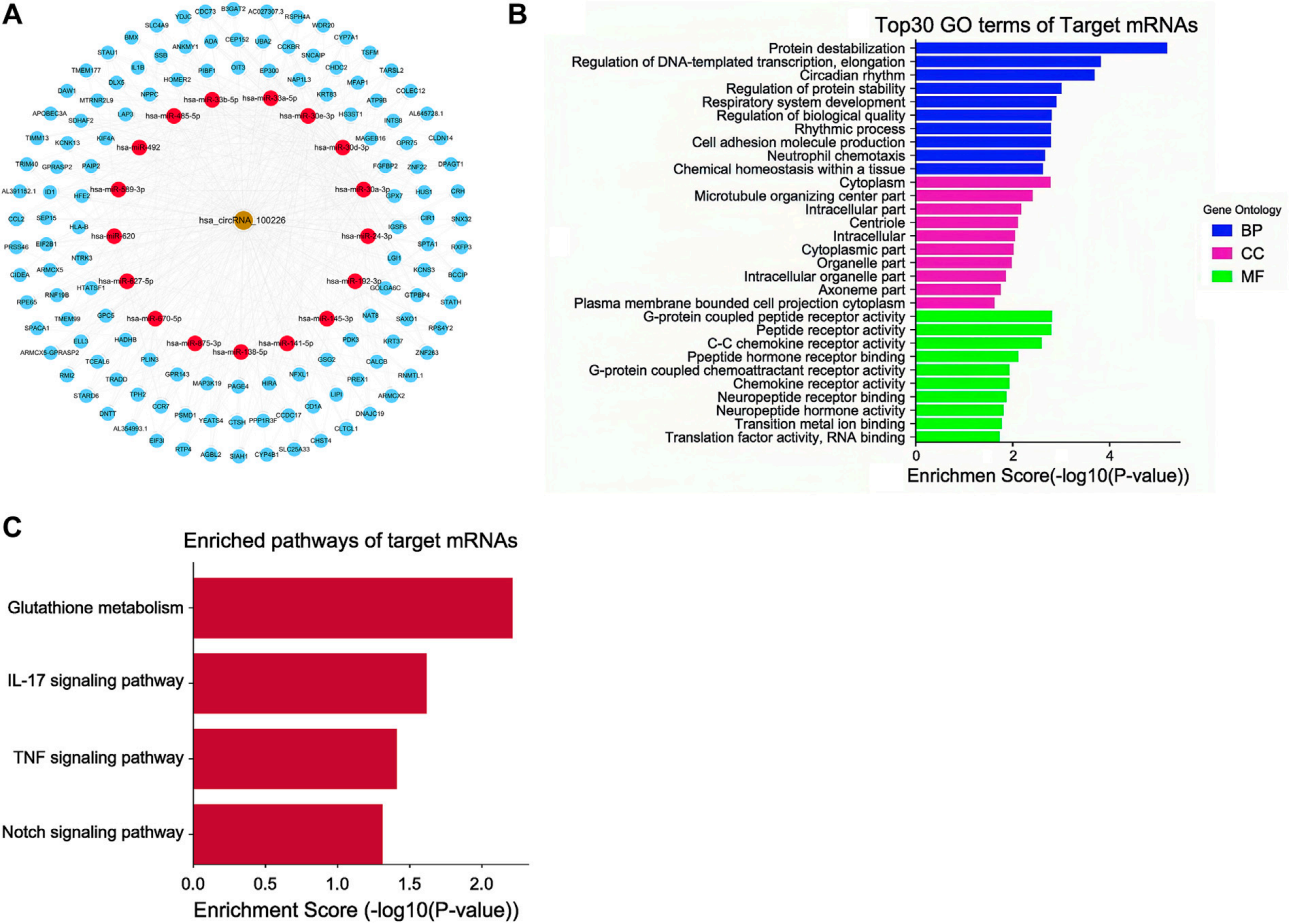
ceRNA analysis of circEPS15. **(A)** CeRNA network plot of circEPS15: the gold circle represents circEPS15 (circRNA-100226), red circle represents miRNA, and blue circle represents mRNA. A ceRNA network including 1 circRNA, 17 miRNAs, and 127 mRNAs was constructed by integrated analysis. **(B)** GO analysis of ceRNA target genes. The top 30 GO terms of target mRNAs and their biological process (BP), cellular component (CC), and molecular function (MF) categories identified by DAVID. **(C)** KEGG analysis of ceRNA target genes. The statistically significant KEGG pathways of target mRNAs identified by DAVID.

circRNAs act as microRNA sponges associated with related miRNAs, and together, they make up the circRNA-miRNA axis involved in disease pathogenesis. To determine the function of circEPS15, we constructed a circEPS15-miRNA-mRNA network to identify the potential targets of circEPS15 (Figure 5A). Detailed information on circEPS15-miRNA-mRNAs is shown in Supplementary Table S3. GO and KEGG analysis of the target mRNAs of circEPS15 was performed. The GO analysis indicated that the BPs were protein destabilization, regulation of DNA-templated transcription, elongation and circadian rhythm. The top three GO terms in the molecular function (MF) category were G-protein coupled peptide receptor activity, peptide receptor activity and C-C chemokine receptor activity. The top three GO terms in the cellular component (CC) category were cytoplasm, microtubule organizing center part and intracellular part (Figure 5B). Additionally, KEGG analysis indicated the related pathways and associated functions. Four KEGG pathways for the target genes of circEPS15 were glutathione metabolism, the IL-17 signaling pathway, the TNF signaling pathway and the Notch signaling pathway, indicating the role of circEPS15 in HCC (Figure 5C). In the prediction, the top five miRNAs for circEPS15 sponging were hsa-miR-24-3p, hsa-miR-138-5p, hsa-miR-145-3p, hsa-miR-620 and hsa-miR-875-3p (Supplementary Figures S3A). Additionally, DIANA-miRPath analysis revealed that these five miRNAs were related to ECM-receptor interactions, the cell cycle and protein binding transcription factor activity (Supplementary Figures S3B,C).

### Evaluation of the circEPS15 Protein-Coding Ability

**FIGURE 6:**
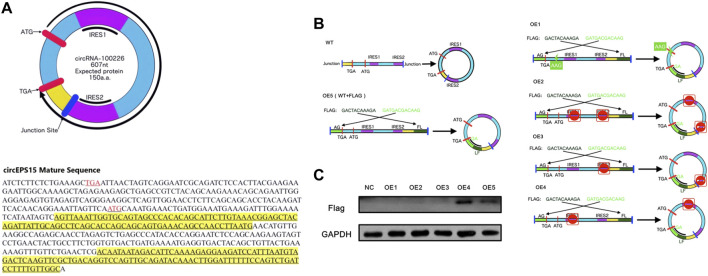
Evaluation of the protein coding ability of circEPS15. **(A)** Upper panel: The putative open reading frame (ORF) in circEPS15. Note that the circEPS15 junction is inside the ORF. Lower panel: The sequences of the putative ORF are shown in black, internal ribosomal entrance site (IRES) sequences are shown in yellow, and start (ATG) and stop (TGA) codons are shown in red. **(B)** WT: Illustration showing how endogenous circEPS15 is formed; the circular junction is inside the ORF and forms unique sequences (yellow). OE5: A FLAG tag was divided on both sides (light and dark green). Circularization of this vector formed the same circular RNA as endogenous circEPS15, except with a FLAG tag added behind the ORF. OE1: The start codon was mutated from ATG to AAG to clarify the role of the start codon. OE2: IRES sequences (IRES1 and IRES2) in circEPS15 were truncated. OE3: IRES sequences (IRES2) in circEPS15 were truncated. OE4: IRES sequences (IRES1) in circEPS15 were truncated. **(C)** A FLAG tag antibody was used to detect circEPS15 expression in 293T cells transfected with the vectors described above.

The circEPS15 sequence was matched in circRNADb; therefore, we next analyzed the putative open reading frame (ORF) in circEPS15 (Figure 6A). There was a potential spanning junction ORF in circEPS15 that could encode a 150-amino acid protein. Conservation analysis showed that this ORF was highly conserved among different species, suggesting that this ORF was translatable. To test the putative ORF transcriptional ability, we cloned wild-type (ATG) or mutant-type (AAG) circEPS15 and inserted it into the GV486 vector (Figure 6B). To test the putative IRES activity in circEPS15, we also cloned a full-length or truncated putative circEPS15 IRES into the GV486 vector. To add a FLAG tag to the gene of interest, we moved the junction to the stop codon, with side flanking sequences (circEPS15-FLAG) (Figure 6B). We transfected these plasmids into 293T cells and detected their potential translated products. As shown in Figure 6C, the FLAG antibody only detected an ∼25 kDa protein in the wild-type circEPS15-FLAG (OE5) and circEPS15 (ins_FLAG)-IRES1del (OE4)-transfected cells but not in the mutated (AAG), IRES2 truncated, or IRES1 and IRES2 truncated circEPS15-FLAG transfected cells. The scores for the IRES prediction in these five sequences were consistent with the western blotting results, wherein OE4 and OE5 had the highest scores (0.75) by IRESFinder (Table 2). These results indicated that circEPS15 can encode a protein, the ORF is translatable, and ATG and IRES2 are essential for the 5′-cap-independent translation of circEPS15.

**TABLE 2:** The IRESfinder was used to identify the IRES sequence in different circRNAs. The region with the highest score was considered to be the IRES sequence for the circRNA.

		ORF (n)	Score (max)	Score (mean)	Score (sd)
OE1	circEPS15(ins_FLAG)-AAG	—	—	—	—
OE2	circEPS15(ins_FLAG)-IRES1/2del	4	0.60	0.55	0.03
OE3	circEPS15(ins_FLAG)-IRESdel2	7	0.72	0.59	0.07
OE4	circEPS15(ins_FLAG)-IRES1del	6	0.75	0.59	0.08
OE5	circEPS15(ins_FLAG)	9	0.75	0.6	0.08

## Discussion

Because various studies have shown that many circRNAs can be stably expressed in eukaryotic cells, several researchers have investigated the role of circRNAs in various diseases. The role(s) of circRNAs in tumors is also becoming a hot research topic.

At present, research on the role of circRNAs in tumors is mainly focused on the discovery and identification of new target circRNAs, their influence on tumor biological behavior, and the molecular mechanism of circRNAs.

A variety of new circRNAs have been found due to the popularization and application of circRNA chips. Through RNA enzyme digestion and sequencing technology, we can identify the circular structure of newly found circRNAs and confirm them as circRNAs.

The role of circRNAs in tumor biological behavior is becoming a new research hotspot. In hepatocellular carcinoma, some circRNAs have been found and confirmed to be related to the biological behavior and prognosis of hepatocellular carcinoma. For example, circFBLIM1 can competitively bind to miR-346 through a ceRNA mechanism, thus promoting the progression of HCC. This study indicates that circRNAs exist in HCC and play an important role in the occurrence and development of HCC. As a new type of RNA, circRNA should be investigated to confirm its role in liver cancer, as it may increase detection of liver cancer and provide a novel way(s) to treat the disease.

The molecular mechanism of circRNA has always been an interesting and difficult area in this field. Interestingly, artificial circRNA has been proven to be translatable in eukaryotic cells (Chen and Sarnow, 1995), and the current evidence also shows that other types of noncoding RNAs can initiate protein synthesis, raising the question of whether endogenous circRNA can encode proteins in mammalian cells (Magny et al., 2013; Lauressergues et al., 2015; Nelson et al., 2016). At present, the molecular mechanisms of circRNAs are miRNA sponge adsorption, ceRNA mechanisms, and recently discovered and confirmed coding polypeptide effects in some circRNAs and lncRNAs. For example, circFBXW7, found in glioma, can encode small peptides and inhibit the growth and replication of glioma, and peptides encoded by a segment of lncRNA are related to SERCA activity in muscle. These findings greatly expand the possible mechanism of circRNAs and overturn the belief that circRNAs cannot be translated as noncoding RNAs. Protein, as the basic component of life, is an important form of biological function.

In this study, we detected the expression level of circEPS15, statistically analyzed the overall survival of corresponding liver cancer patients and selected the effective cutoff value, divided circEPS15 into a high expression group and a low expression group, and found that this molecule from eps15 was related to the overall survival of patients. This conclusion suggests that circEPS15 can be used as a potential biomarker for prognostic analysis and monitoring of HCC patients.

In this study, we used microarray and qRT-PCR analyses, combined with *in vivo* experiments, to determine the relationship between the downregulation of circEPS15 expression in HCC and the prognosis of HCC. The downregulation of circEPS15 expression in HCC tissues was significantly correlated with tumor differentiation and intravascular tumor thrombus, suggesting that circEPS15 has an antitumor effect and may play an important role in the epithelial-mesenchymal transition of HCC. This hypothesis has been verified *in vivo* and *in vitro*. In addition, we demonstrated that circEPS15 can regulate mRNA through the ceRNA regulatory network and has the ability to encode proteins.

CircEPS15 is derived from the parent gene eps15. Therefore, we need to understand eps15 and its functions before elucidating the functions of circEPS15. Eps15 is transcribed from ch38. p13, which encodes a component of the EGFR pathway (Niehof and Borlak, 2008), exists in the pits of the reticular protein coating, and participates in the receptor-mediated endocytosis of epidermal growth factor (EGF) (Morgan et al., 2003). In addition, alternative splicing leads to multiple transcriptional variants that encode different subtypes (Kieken et al., 2010). In addition to affecting cell migration and cancer-related signal transduction, eps15 is also associated with poor prognosis in various tumor types, including breast cancer, esophageal squamous cell carcinoma, and glioma (Smith et al., 2000; Li et al., 2013; Shi et al., 2015). Previous studies have shown that edh2 (eps15 homologous domain contains 2) may inhibit the migration and invasion of hepatocellular carcinoma through interactions with E-cadherin (Liu et al., 2016). However, the underlying mechanism by which eps15 inhibits tumors remains unclear. Consistent with previous studies, the mRNA expression of eps15 was significantly downregulated in HCC tissues compared to matched nontumor tissues, while the eps15 mRNA level in HCC tissues was positively correlated with that of circEPS15. These observations suggest that the expression of circEPS15 in HCC may be mainly regulated by host gene amplification. Bai et al. (2018) and He et al. (2017) reported that circFBLIM1 and circGRFA1 can regulate the corresponding linear fblim1 and grfa1 mRNA transcriptional expression through a ceRNA mechanism. Therefore, circEPS15 may regulate the transcriptional expression of the corresponding linear eps15 mRNA through the abovementioned ceRNA mechanism. This hypothesis will need to be further tested, proven and discussed in future research.

In this study, we found that circEPS15 could encode a protein *in vitro* by matching the RNA deep sequencing data with circaDB. Given the key role of the eps15 gene in tumorigenesis, we hypothesized that the eps15 subtype with the same function and the shorter and more stable translated protein of circEPS15 may be key regulators of cell proliferation. As this study is limited to cultured cells, the clinical importance of circEPS15 needs to be studied in a larger clinical cohort. In addition, this study confirmed the translatability of circEPS15, but the structure and function of the peptide encoded by circEPS15 need to be further verified by synthesizing specific antibodies, which are expected in subsequent studies. As an important biological regulator, circRNAs have been found to encode polypeptides in gliomas. However, no translatable circRNAs or their products have been reported in HCC.

## Conclusion

A novel circRNA named circEPS15 was found in HCC, and its expression level was related to the overall survival of HCC patients. Our observations of the expression and activity of circEPS15 suggest that circEPS15 and its translatability may play some role in the progression of HCC and the clinical prognosis of patients.

## Data Availability

The original contributions presented in the study are publicly available. This data can be found here: GSE189043.

## Abbreviations

HCC, Hepatocellular carcinoma; circRNAs, Circular RNAs; IRES, Internal ribosomal entry site; TCGA, The Cancer Genome Atlas; RT-PCR, Real-time PCR; EMT, Epithelial mesenchymal transition; siRNA, Small interfering RNA; lncRNA, Long non-coding RNA; HCV, Hepatitis C virus; EPS15, Epidermal growth factor receptor pathway substrate 15; FC, Fold-change; cDNAs, Complementary DNAs; ROC, Receiver Operating Characteristic; AUC, Area Under Curve; NC, Negative control; EGF, Epidermal growth factor; EDH2, Eps15 homology domain-containing 2.
