# Supplementary figures and images for: circEPS15 Overexpression in Hepatocellular Carcinoma Modulates Tumor Invasion and Migration
Source: Front Genet. 2022 Feb 8;13:804848. doi: 10.3389/fgene.2022.804848 (PMC8861492; doi:10.3389/fgene.2022.804848)

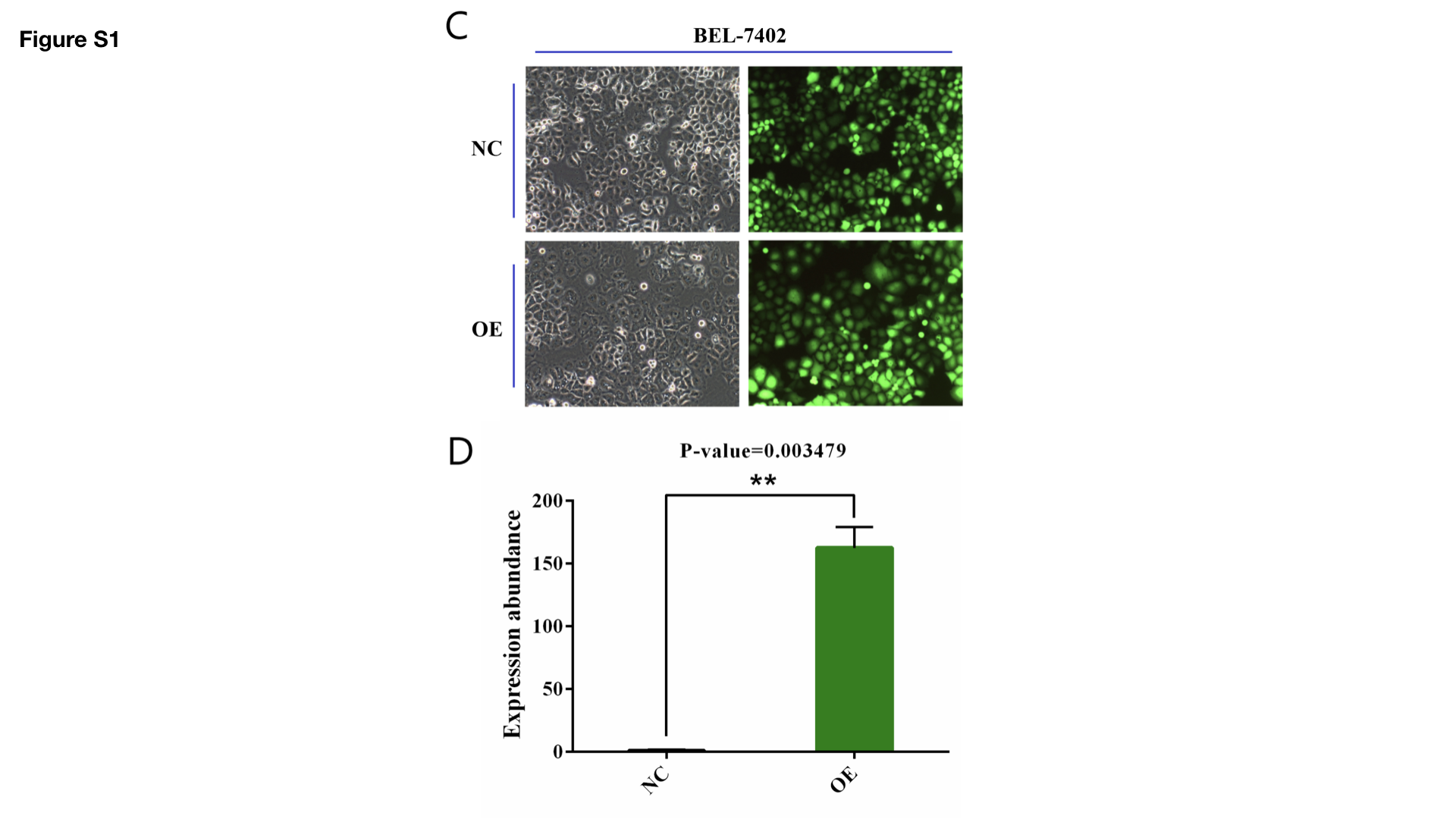

Supplement: Supplementary file 1 [file Image3.TIFF]

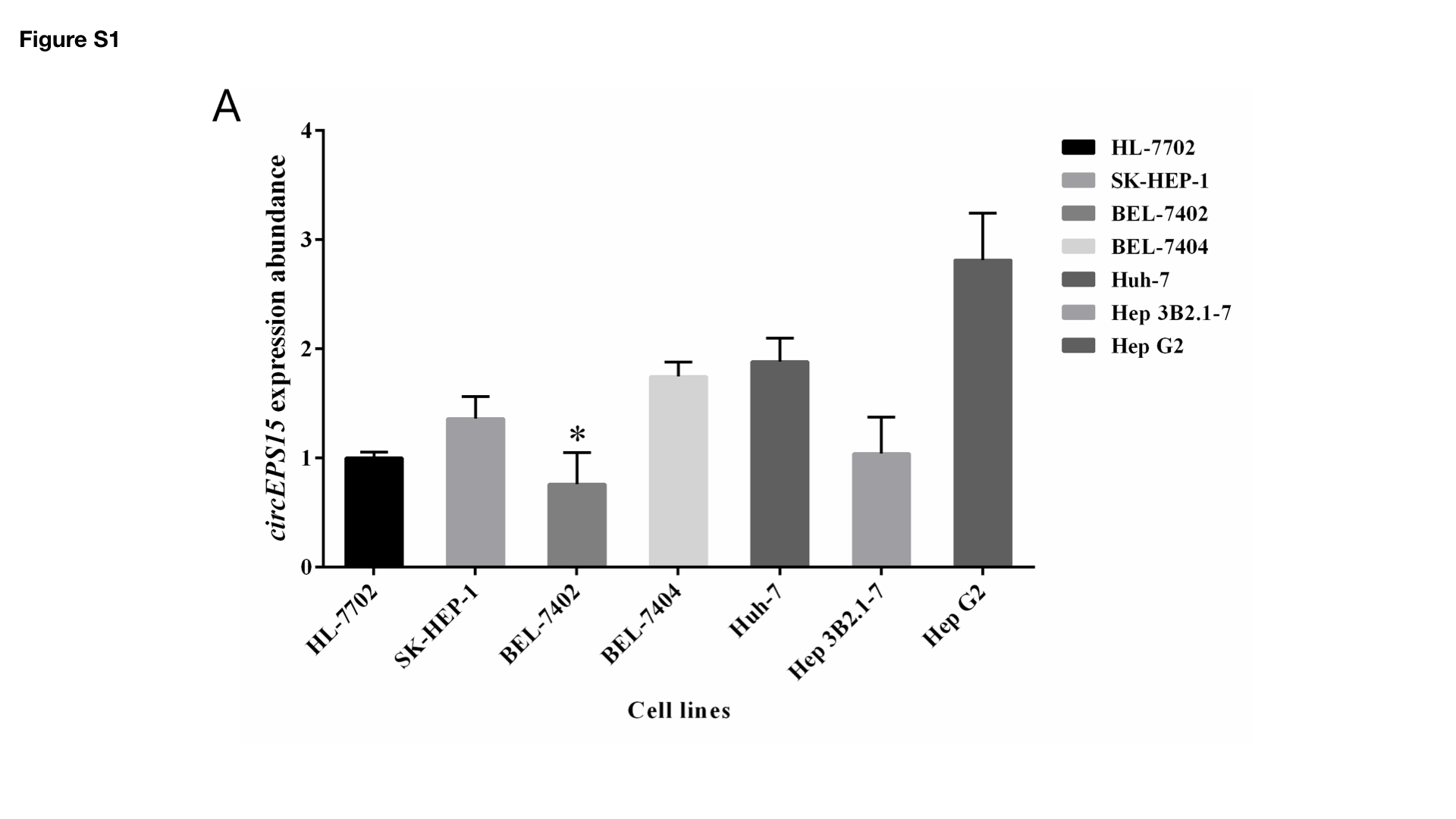

Supplement: Supplementary file 2 [file Image1.TIFF]

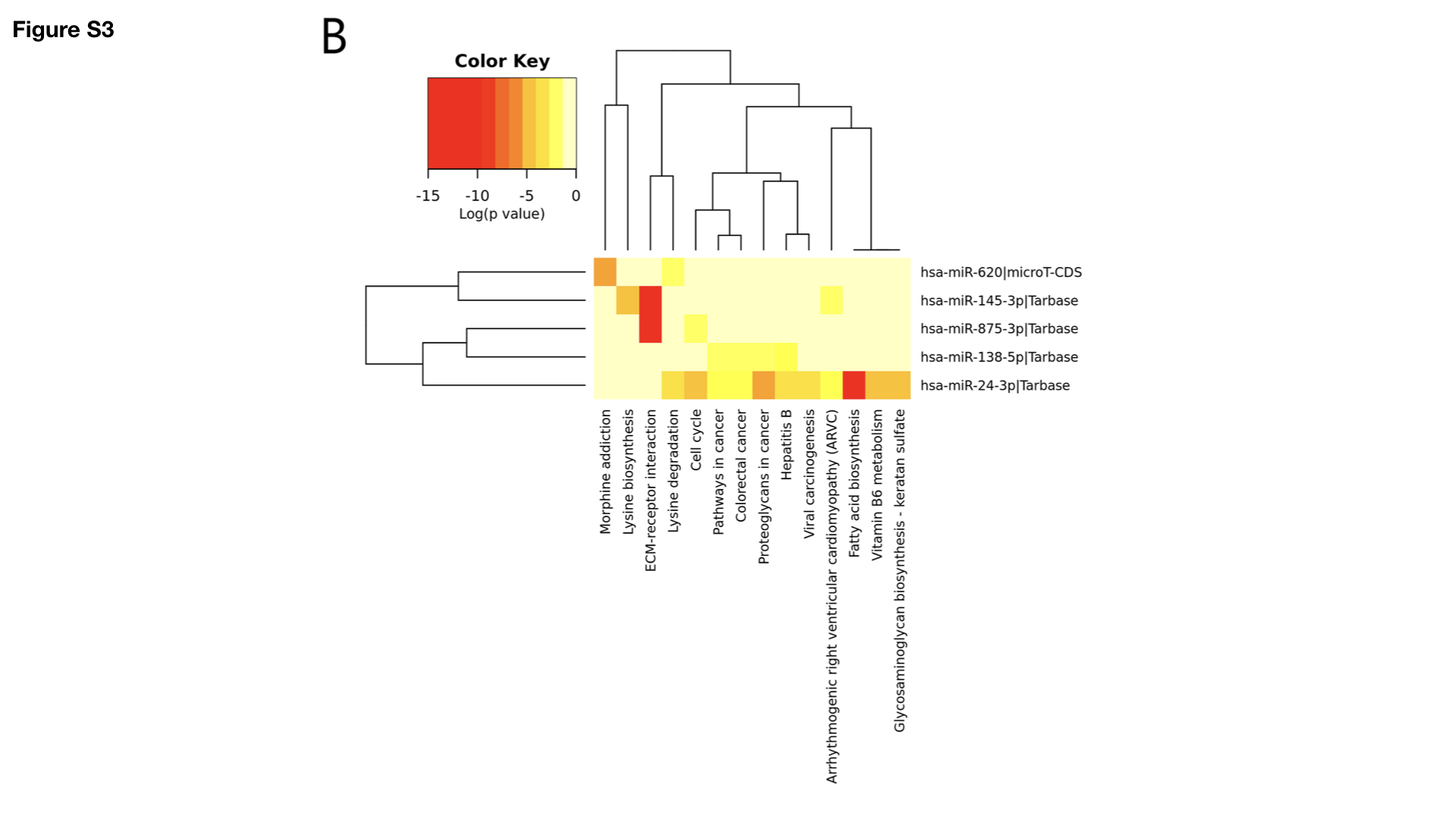

Supplement: Supplementary file 3 [file Image9.TIFF]

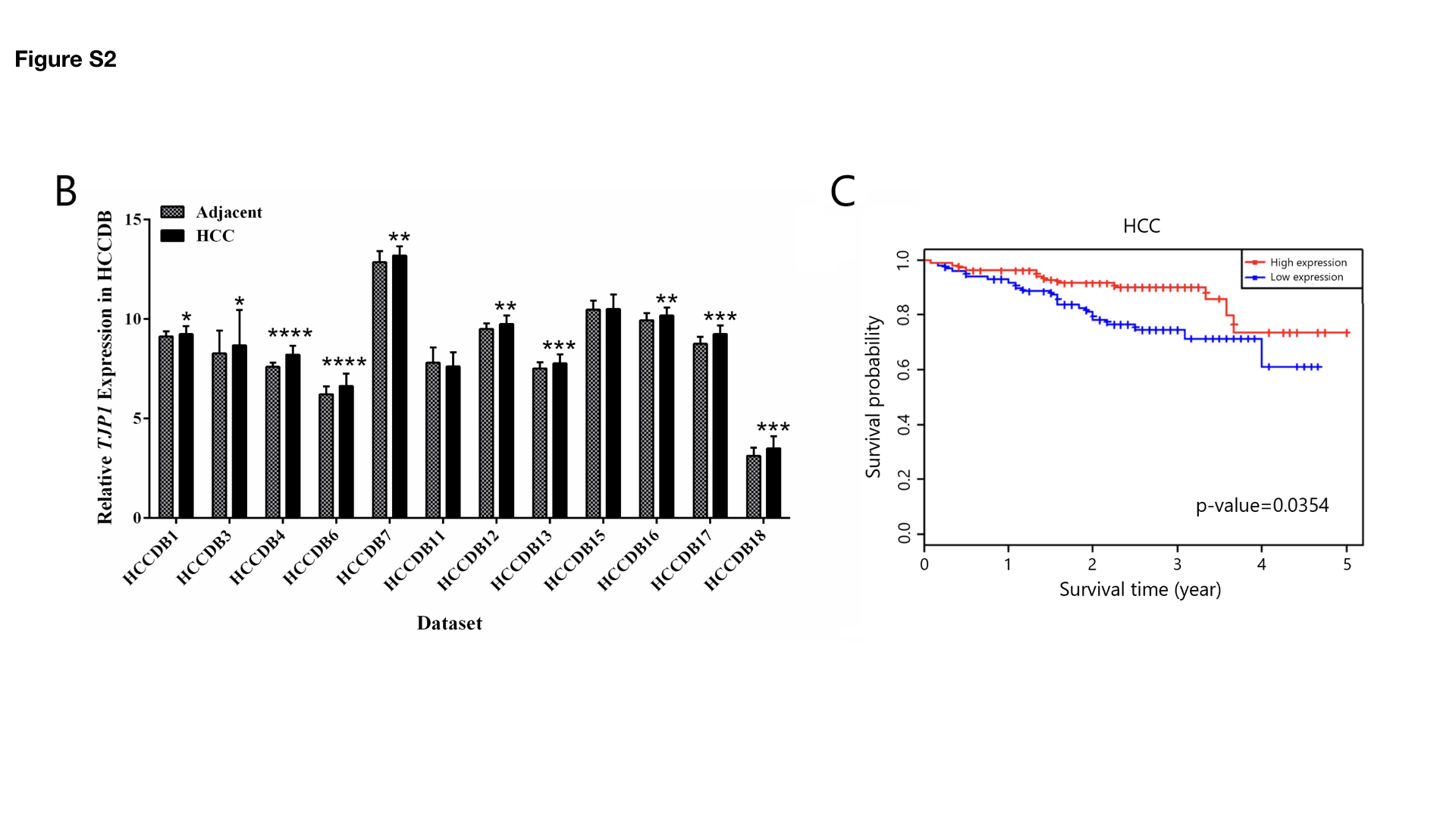

Supplement: Supplementary file 4 [file Image5.TIFF]

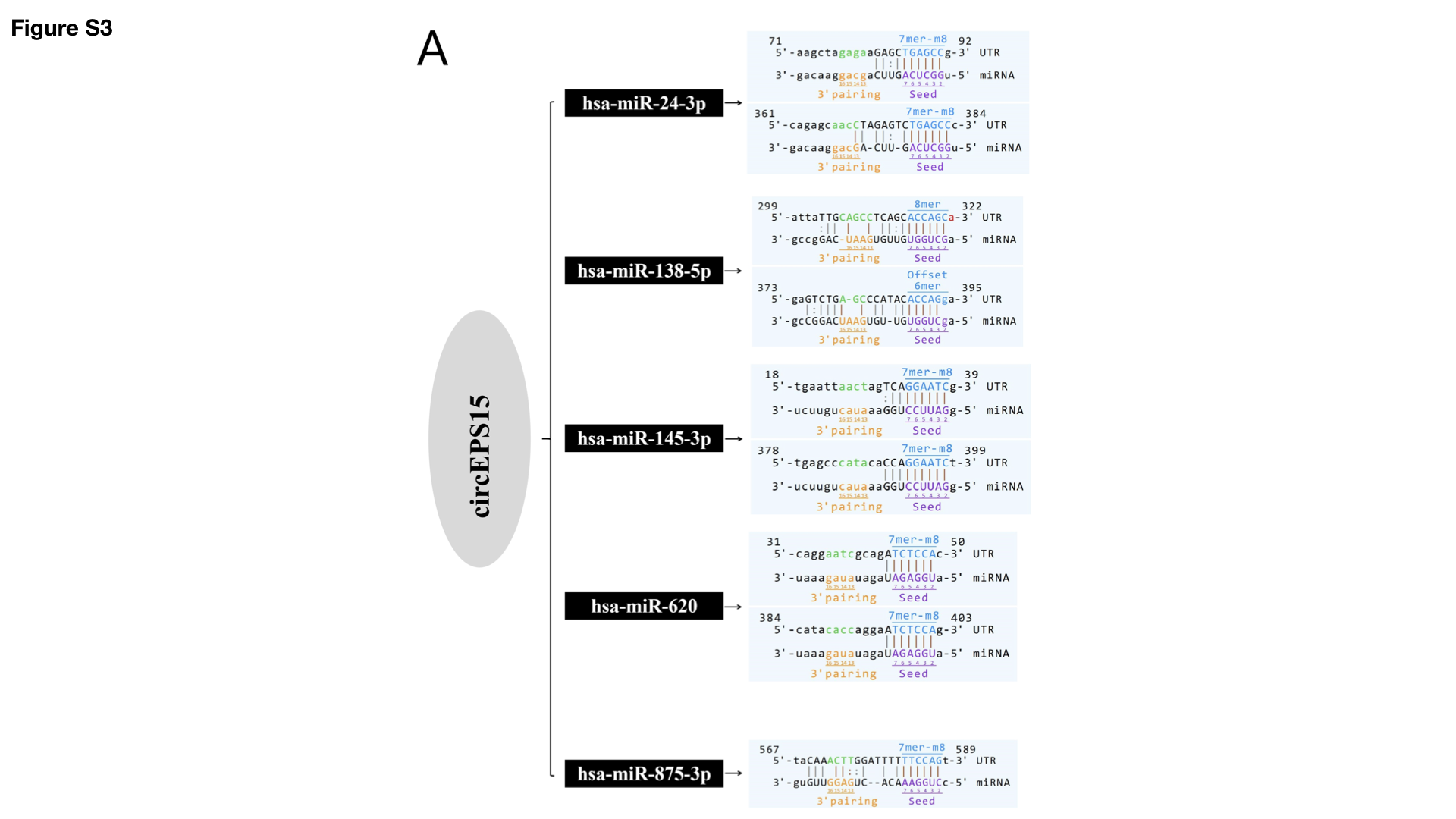

Supplement: Supplementary file 5 [file Image8.TIFF]

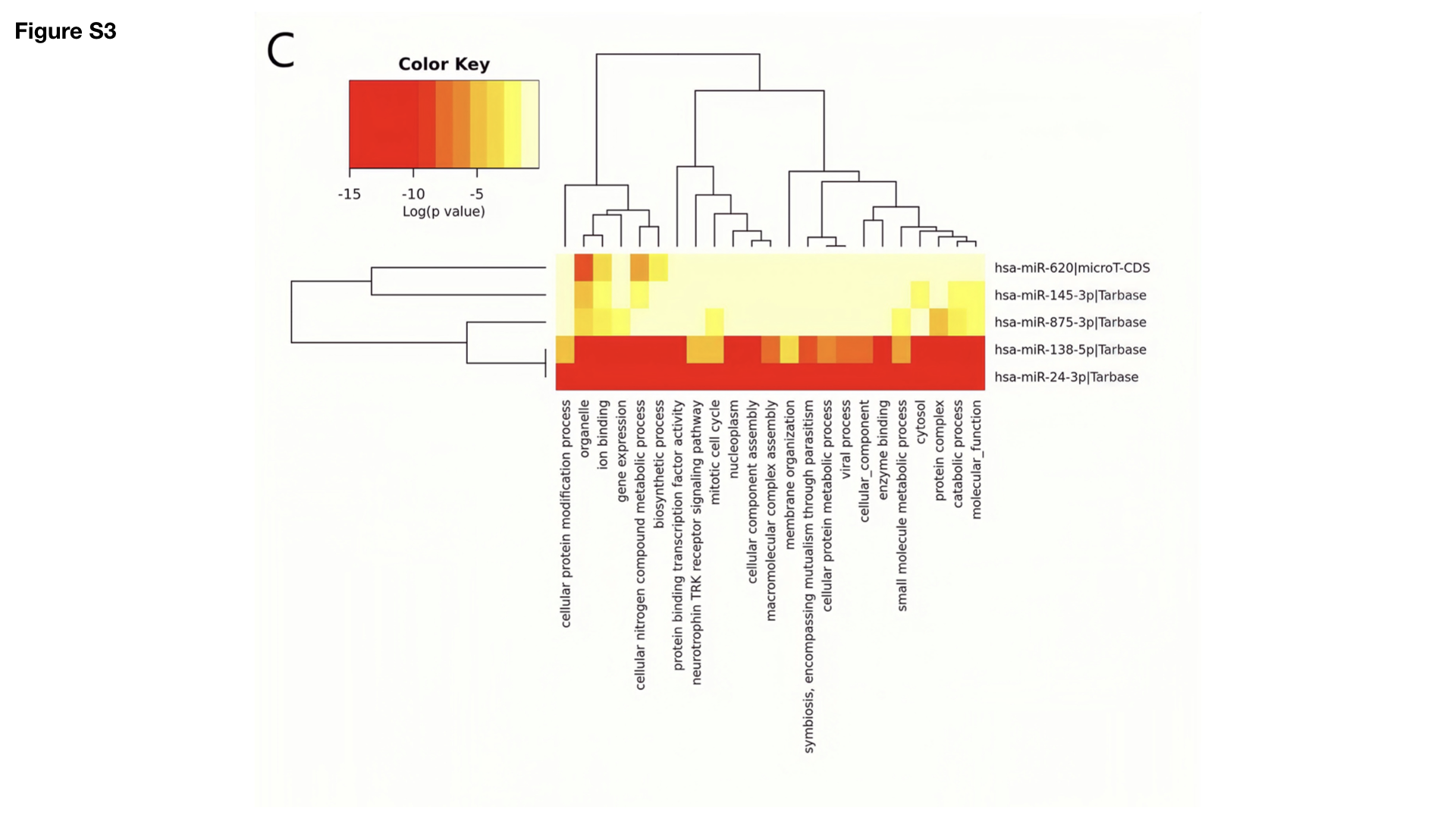

Supplement: Supplementary file 7 [file Image10.TIFF]

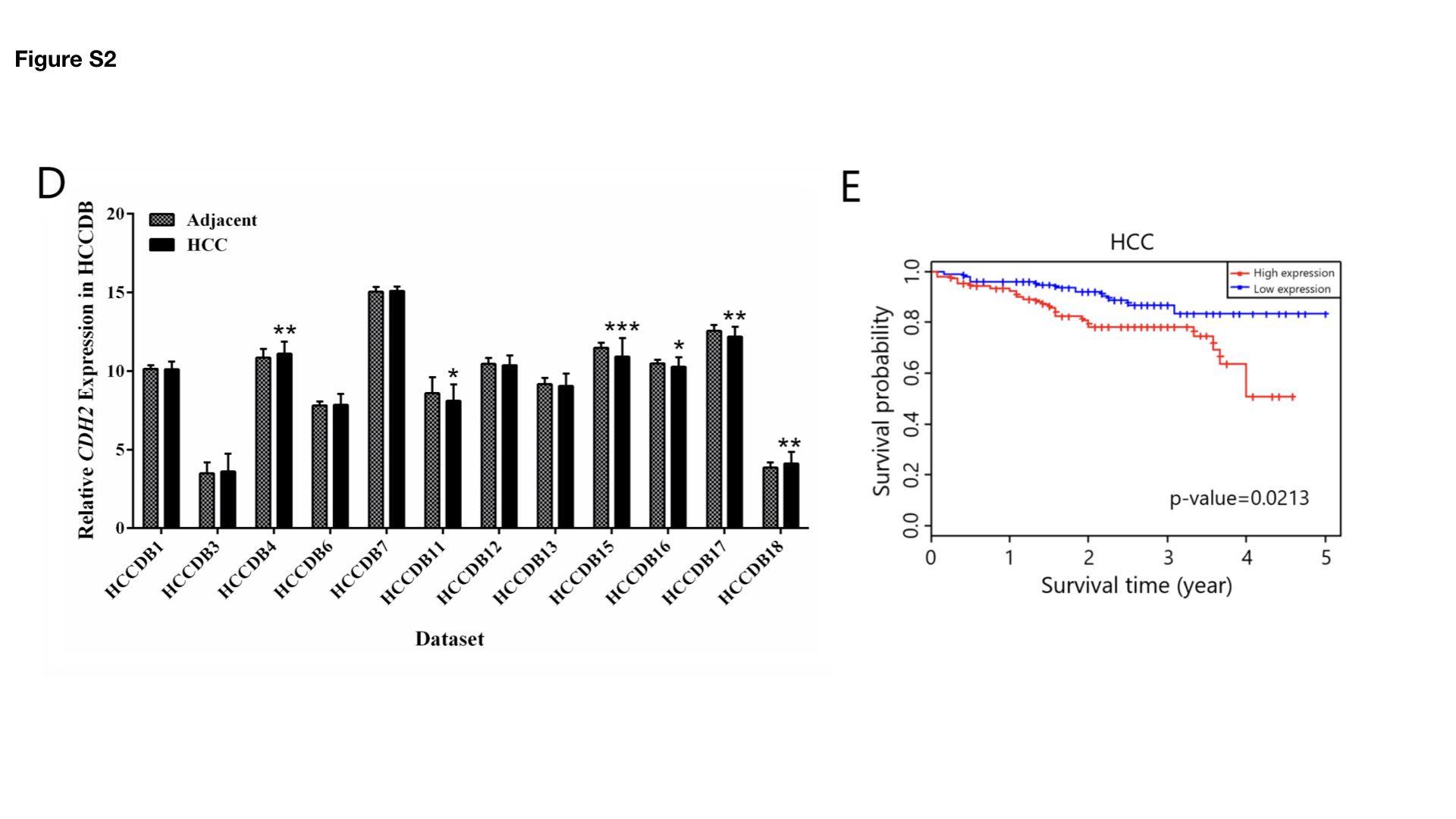

Supplement: Supplementary file 8 [file Image6.TIFF]

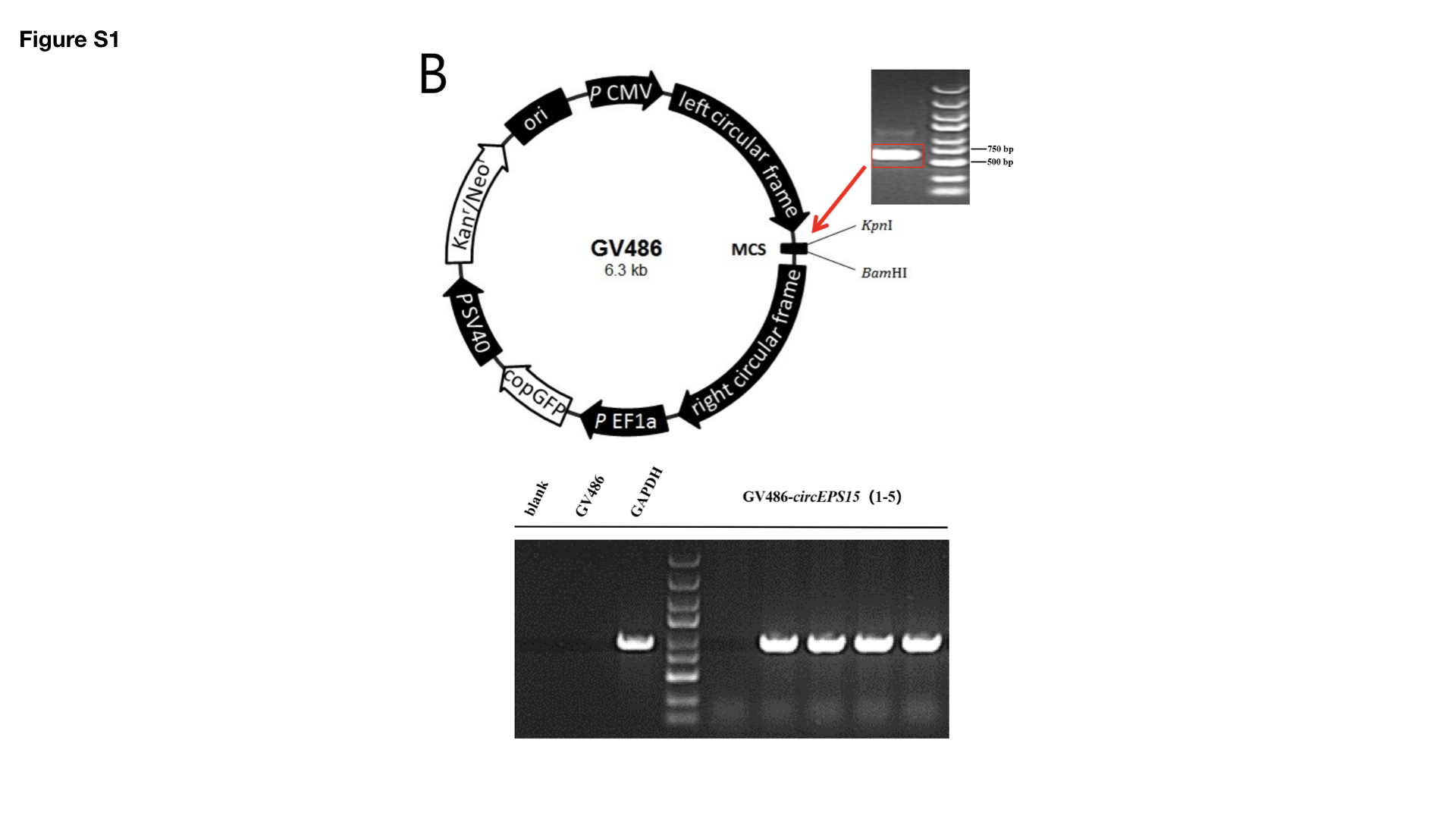

Supplement: Supplementary file 9 [file Image2.TIFF]

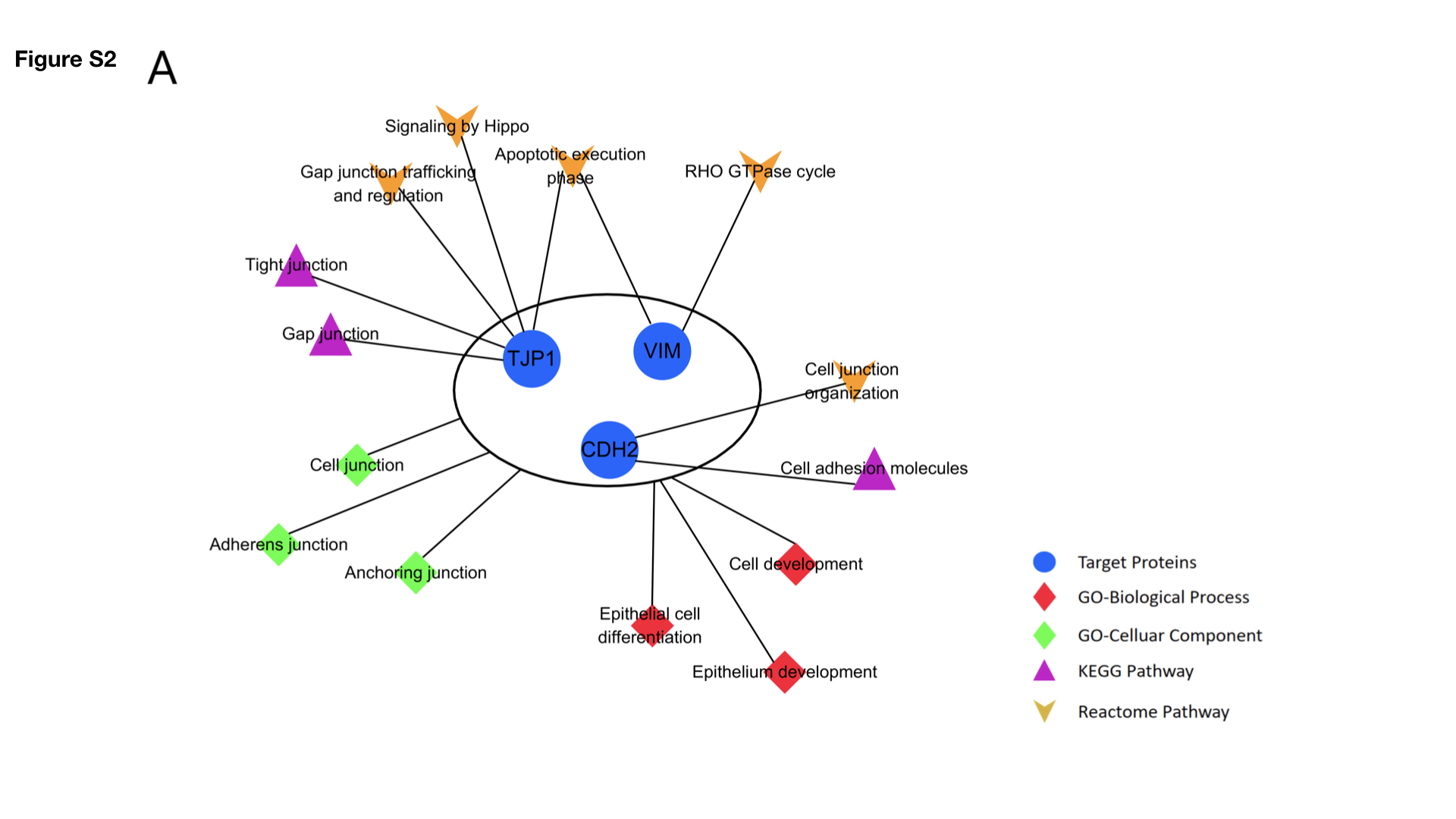

Supplement: Supplementary file 10 [file Image4.TIFF]

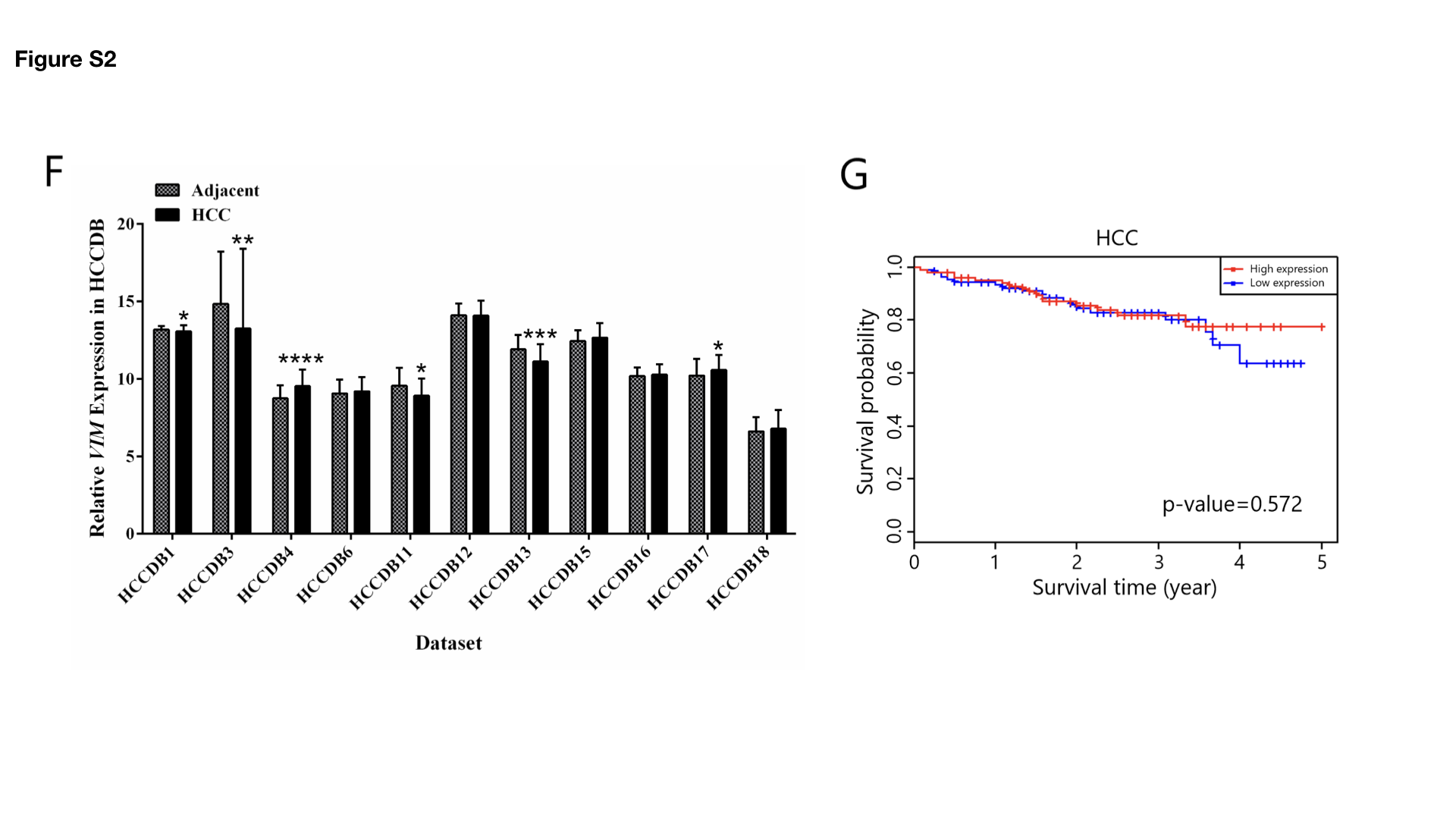

Supplement: Supplementary file 11 [file Image7.TIFF]
