# Supplementary material for: circEPS15 Overexpression in Hepatocellular Carcinoma Modulates Tumor Invasion and Migration
Source: Front Genet. 2022 Feb 8;13:804848. doi: 10.3389/fgene.2022.804848 (PMC8861492; doi:10.3389/fgene.2022.804848)
Supplement: Supplementary file 6 [file DataSheet1.PDF]

Figure S1

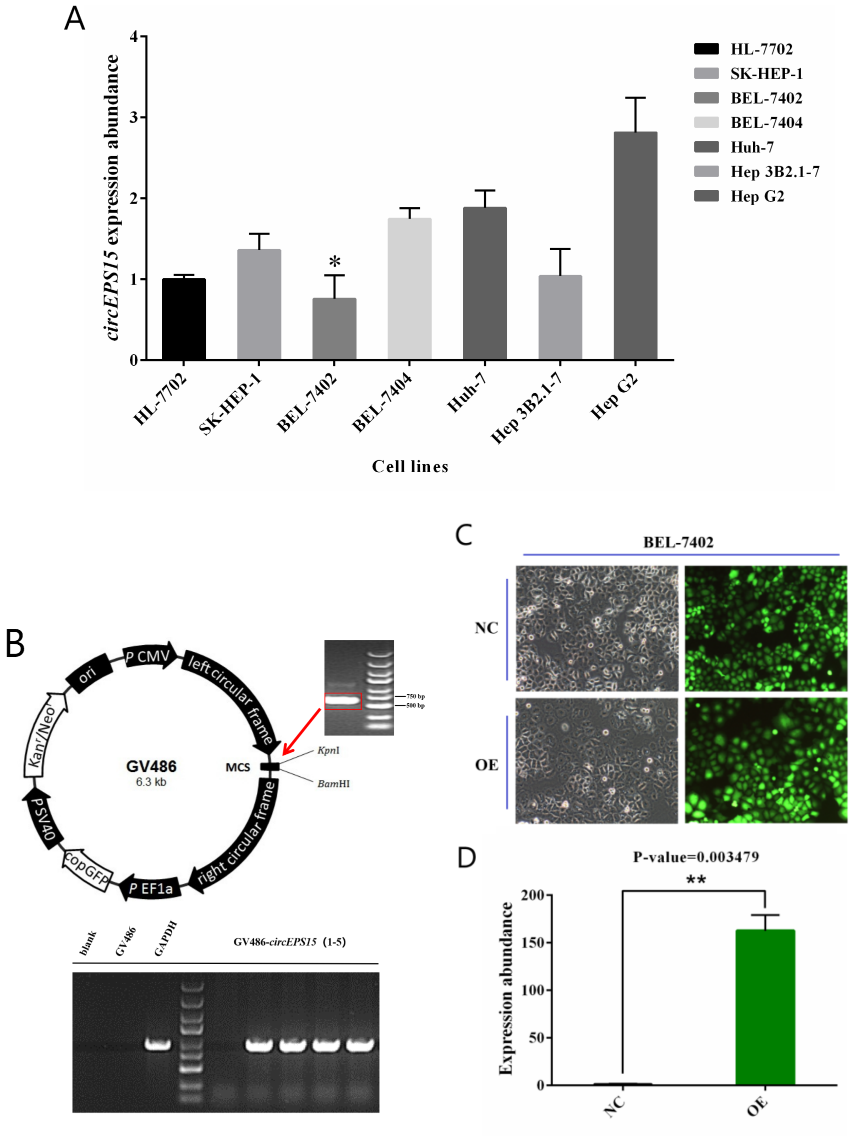

Figure S1. Overexpression of *circEPS15* in BEL7402 cells. (A) Expression of circEPS15 in different cell lines associated with HCC. (B) Schematic diagram of the construction of GV486-ciR vector carrying mature sequence of circEPS15. The entire sequence of mature circEPS15 containing Kpn I/ BamH I restriction sites was subcloned into GV486-ciR vector. The BEL7402 cells were transfected with GV486-ciR (GV486) or circEPS15 (GV486-circEPS15) and cultured under normal conditions. The BEL7402 cells without transfections were used as the negative control. (C) The efficiency of circEPS15 overexpression in BEL7402 cells. GV486-ciR vector containing circEPS15 was transfected into BEL7402 cells (circEPS15) and cultured for 48h. Fluorescence Microscope was used to detect the GFP fluorescence intensity. qRT-PCR was performed to identify circEPS15 expression. BEL7402 cells transfected with GV486-ciR (GV486) were served as negative control (n=3 individual experiments). Comparisons of data were acquired by unpaired t-test.

Figure S2

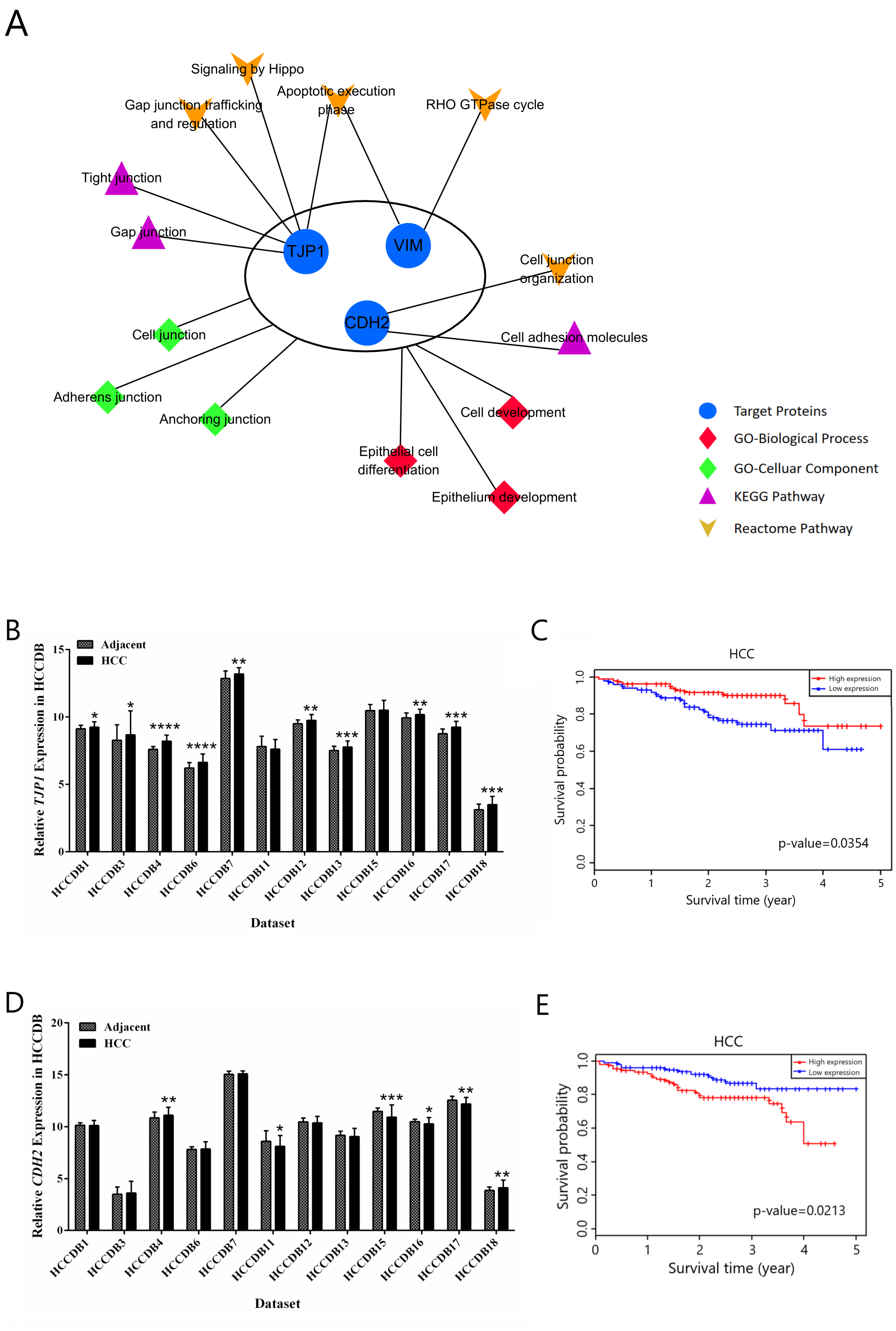
A
Signaling by Hippo
Apoptotic execution phase
Gap junction trafficking and regulation
RHO GTPase cycle
Tight junction
Gap junction
TJP1
VIM
CDH2
Cell junction organization
Cell junction
Cell adhesion molecules
Adherens junction
Anchoring junction
Cell development
Epithelial cell differentiation
Epithelium development
Target Proteins
GO-Biological Process
GO-Celluar Component
KEGG Pathway
Reactome Pathway
B
Adjacent
HCC
Relative TJP1 Expression in HCCDB
HCCDB1
HCCDB3
HCCDB4
HCCDB6
HCCDB7
HCCDB11
HCCDB12
HCCDB13
HCCDB15
HCCDB16
HCCDB17
HCCDB18
Dataset
C
HCC
High expression
Low expression
p-value=0.0354
Survival probability
Survival time (year)
D
Adjacent
HCC
Relative CDH2 Expression in HCCDB
Dataset
E
HCC
High expression
Low expression
p-value=0.0213
Survival probability
Survival time (year)

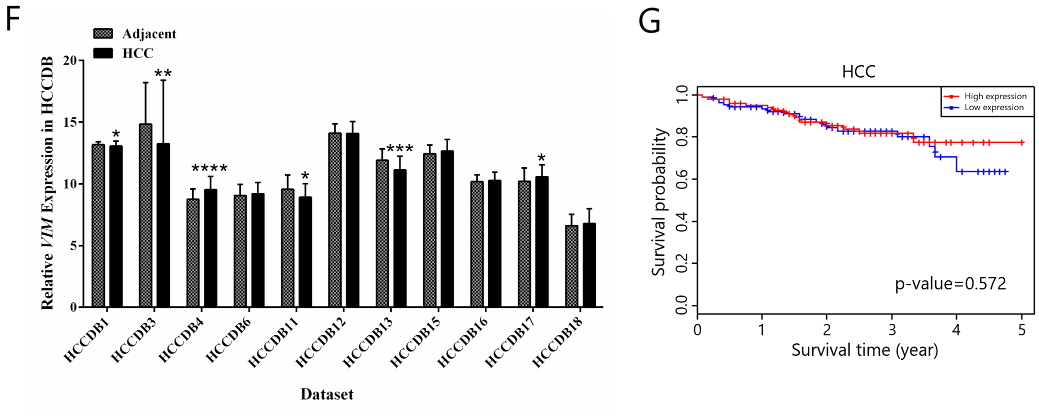

Figure S2. Analysis of TJP1, CDH2 and VIM associated with cell migration and invasion. (A) Biological Function and pathway of TJP1, CDH2 and VIM. (B) Expression and diagnostic value of TJP1, CDH2 and VIM in HCCDB.

Figure S3

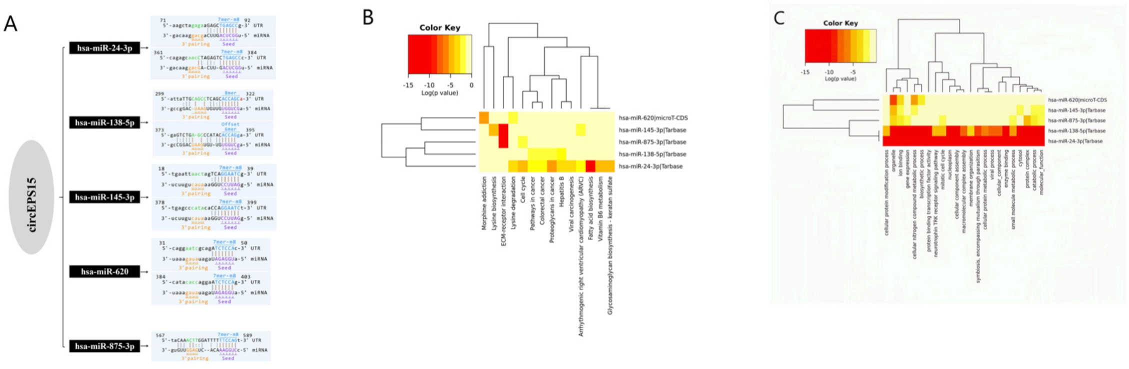

Figure S3. The prediction of the top five MREs for circEPS15 sponging were hsa-miR-24-3p, hsa-miR-138-5p, hsa-miR-145-3p, hsa-miR-620 and hsa-miR-875-3p (A). DIANA-miRPath analysis revealed that these five miRNAs were related to the ECM-receptor interaction, cell cycle and protein binding transcription factor activity (B and C).

Table S1: The detailed information of Top10 up and down-regulated DE circRNAs with one group raw intensity >1000.

circRNA	circBase Name	P-value	Fold Change	Regulation	circRNA_type	chrom	strand	txStart	txEnd	best_transcript	GeneSymbol
hsa_circRNA_103595	hsa_circ_0069086	0.001003597	8.2173262	up	exonic	chr4	+	6599924	6602483	NM_015274	MAN2B2
hsa_circRNA_102476	hsa_circ_0007396	0.017323129	6.8864522	up	exonic	chr19	+	17270204	17273932	NM_001130065	MYO9B
hsa_circRNA_104423	hsa_circ_0001721	0.005497707	5.7986577	up	exonic	chr7	+	90355880	90356126	NM_012395	CDK14
hsa_circRNA_102403	hsa_circ_0048234	0.000139632	5.6070008	up	exonic	chr19	+	1417498	1432689	NM_170711	DAZAP1
hsa_circRNA_100477	hsa_circ_0016867	0.01368216	5.1745256	up	exonic	chr1	+	230798886	230807386	NM_007357	COG2
hsa_circRNA_001914	hsa_circ_0000902	6.36731E-05	4.3454272	up	antisense	chr19	+	15510546	15510663	NM_014371	AKAP8L
hsa_circRNA_102534	hsa_circ_0006992	0.009968013	4.2279229	up	exonic	chr19	+	34954930	34957919	NM_005499	UBA2
hsa_circRNA_103803	hsa_circ_0072008	0.005459928	4.1921088	up	exonic	chr5	-	16758226	16764505	NM_012334	MYO10
hsa_circRNA_101835	hsa_circ_0005615	0.011475866	3.8346224	up	exonic	chr16	+	68155889	68157024	NM_004555	NFATC3
hsa_circRNA_100788	hsa_circ_0021650	0.049768956	3.7957717	up	exonic	chr11	+	34107851	34107960	NM_005898	CAPRIN1
hsa_circRNA_103557	hsa_circ_0068631	0.007044189	14.4584201	down	exonic	chr3	-	195802029	195803993	NM_003234	TFRC
hsa_circRNA_101753	hsa_circ_0002696	0.002092014	12.1869516	down	exonic	chr16	+	21623965	21636436	NM_016025	METTL9
hsa_circRNA_103484	hsa_circ_0067597	7.52349E-05	7.9928254	down	exonic	chr3	-	141889166	141911775	NR_033289	GK5
hsa_circRNA_100883	hsa_circ_0005918	0.000785233	7.5499757	down	exonic	chr11	-	72695132	72700142	NM_014824	FCHSD2
hsa_circRNA_100946	hsa_circ_0024162	0.001463835	7.3816772	down	exonic	chr11	-	107673726	107673881	NM_017515	SLC35F2
hsa_circRNA_104227	hsa_circ_0078297	0.001665858	5.9467732	down	exonic	chr6	+	151336015	151336829	NM_001242767	MTHFD1L

hsa_circRNA_100226	hsa_circ_0005567	0.00092488	5.8073311	down	exonic	chr1	-	51868106	51874004	NM_001981	EPS15
hsa_circRNA_103454	hsa_circ_0067103	3.83182E-06	5.5282321	down	exonic	chr3	-	125032151	125032500	NM_021964	ZNF148
hsa_circRNA_103627	hsa_circ_0069559	0.004910528	4.8329669	down	exonic	chr4	-	40892380	40895428	NM_004307	APBB2
hsa_circRNA_104050	hsa_circ_0075504	0.00405856	4.7223535	down	exonic	chr6	-	3410421	3416089	NM_015482	SLC22A23

Table S2. The primers sequence of related qPCR validation

Gene	Primer sequence
β-actin	F:5'GTGGCCGAGGACTTTGATTG3'<br>R:5’CCTGTAACAACGCATCTCATATT3’
circEPS15	F:5'TCCAGTCTGATCCTTTTGTTGG3’<br>R:5’CTGTTTCTTGCTGTAGACGGCT3’

Table S3. The detailed information of circEPS15-miRNAs-mRNAs

SeqName	GeneSymbol	CeNames	CeSymbols	P-values	CommonMirnas
circEPS15	hsa_circ_0005567	NM_000183	HADHB	0.00000028	hsa-miR-30e-3p,hsa-miR-670-5p,hsa-miR-589-3p,hsa-miR-30a-3p,hsa-miR-33a-5p,hsa-miR-33b-5p
circEPS15	hsa_circ_0005567	NM_001164404	GOLGA6C	0.00000129	hsa-miR-875-3p,hsa-miR-30d-3p,hsa-miR-30e-3p,hsa-miR-589-3p,hsa-miR-30a-3p,hsa-miR-33a-5p,hsa-miR-33b-5p
circEPS15	hsa_circ_0005567	NM_001838	CCR7	0.00002238	hsa-miR-620,hsa-miR-627-5p,hsa-miR-30d-3p,hsa-miR-30e-3p,hsa-miR-670-5p,hsa-miR-30a-3p
circEPS15	hsa_circ_0005567	NM_004538	NAP1L3	0.00002835	hsa-miR-30d-3p,hsa-miR-30e-3p,hsa-miR-30a-3p,hsa-miR-33a-5p,hsa-miR-33b-5p
circEPS15	hsa_circ_0005567	NM_014782	ARMCX2	0.00005904	hsa-miR-138-5p,hsa-miR-30d-3p,hsa-miR-30e-3p,hsa-miR-30a-3p
circEPS15	hsa_circ_0005567	NM_006963	ZNF22	0.00006927	hsa-miR-145-3p,hsa-miR-24-3p,hsa-miR-30d-3p,hsa-miR-30e-3p,hsa-miR-30a-3p
circEPS15	hsa_circ_0005567	NM_024529	CDC73	0.00009004	hsa-miR-24-3p,hsa-miR-627-5p,hsa-miR-30d-3p,hsa-miR-30e-3p,hsa-miR-30a-3p
circEPS15	hsa_circ_0005567	NM_004507	HUS1	0.00010208	hsa-miR-30d-3p,hsa-miR-30e-3p,hsa-miR-589-3p,hsa-miR-30a-3p,hsa-miR-485-5p
circEPS15	hsa_circ_0005567	NM_000780	CYP7A1	0.00010855	hsa-miR-875-3p,hsa-miR-30d-3p,hsa-miR-30e-3p,hsa-miR-492,hsa-miR-30a-3p
circEPS15	hsa_circ_0005567	NM_001763	CD1A	0.00011145	hsa-miR-620,hsa-miR-589-3p,hsa-miR-33a-5p,hsa-miR-33b-5p
circEPS15	hsa_circ_0005567	NM_015907	LAP3	0.00012531	hsa-miR-30d-3p,hsa-miR-30e-3p,hsa-miR-30a-3p
circEPS15	hsa_circ_0005567	NM_017453	STAU1	0.00012987	hsa-miR-138-5p,hsa-miR-620,hsa-miR-30d-3p,hsa-miR-30e-3p,hsa-miR-30a-3p
circEPS15	hsa_circ_0005567	NM_152308	RMI2	0.00013472	hsa-miR-589-3p,hsa-miR-485-5p,hsa-miR-33a-5p,hsa-miR-33b-5p
circEPS15	hsa_circ_0005567	NM_017841	SDHAF2	0.00013472	hsa-miR-24-3p,hsa-miR-30d-3p,hsa-miR-30e-3p,hsa-miR-30a-3p
circEPS15	hsa_circ_0005567	NM_004088	DNTT	0.00015154	hsa-miR-670-5p,hsa-miR-33a-5p,hsa-miR-33b-5p
circEPS15	hsa_circ_0005567	NM_178821	DAW1	0.00018111	hsa-miR-30d-3p,hsa-miR-30e-3p,hsa-miR-30a-3p
circEPS15	hsa_circ_0005567	NM_145274	TMEM99	0.00020814	hsa-miR-30d-3p,hsa-miR-30e-3p,hsa-miR-30a-3p,hsa-miR-485-5p
circEPS15	hsa_circ_0005567	NM_078468	BCCIP	0.00021419	hsa-miR-30d-3p,hsa-miR-30e-3p,hsa-miR-30a-3p

circEPS 15	hsa_circ_000556 7	NM_0010123 38	NTRK3	0.00025 096	hsa-miR-30d-3p,hsa-miR-30e-3p,hsa-miR-30a-3p
circEPS 15	hsa_circ_000556 7	NM_000022	ADA	0.00025 096	hsa-miR-30d-3p,hsa-miR-30e-3p,hsa-miR-30a-3p
circEPS 15	hsa_circ_000556 7	NM_005849	IGSF6	0.00029 160	hsa-miR-30d-3p,hsa-miR-30e-3p,hsa-miR-30a-3p
circEPS 15	hsa_circ_000556 7	NM_005221	DLX5	0.00029 160	hsa-miR-30d-3p,hsa-miR-30e-3p,hsa-miR-30a-3p
circEPS 15	hsa_circ_000556 7	NM_002807	PSMD1	0.00029 160	hsa-miR-620,hsa-miR-33a-5p,hsa-miR-33b-5p
circEPS 15	hsa_circ_000556 7	NM_139171	STARD6	0.00035 460	hsa-miR-138-5p,hsa-miR-875-3p,hsa-miR-33a-5p,hsa-miR-33b-5p
circEPS 15	hsa_circ_000556 7	NM_152334	TARSL2	0.00038 033	hsa-miR-875-3p,hsa-miR-30d-3p,hsa-miR-30e-3p,hsa-miR-30a-3p
circEPS 15	hsa_circ_000556 7	NM_012310	KIF4A	0.00043 577	hsa-miR-30d-3p,hsa-miR-30e-3p,hsa-miR-492,hsa-miR-30a-3p
circEPS 15	hsa_circ_000556 7	NM_0010395 67	RPS4Y2	0.00043 577	hsa-miR-627-5p,hsa-miR-492,hsa-miR-192-3p,hsa-miR-141-5p
circEPS 15	hsa_circ_000556 7	NM_005726	TSFM	0.00043 577	hsa-miR-30d-3p,hsa-miR-30e-3p,hsa-miR-589-3p,hsa-miR-30a-3p
circEPS 15	hsa_circ_000556 7	NM_007003	PAGE4	0.00043 837	hsa-miR-30d-3p,hsa-miR-30e-3p,hsa-miR-30a-3p
circEPS 15	hsa_circ_000556 7	NM_198531	ATP9B	0.00052 950	hsa-miR-30d-3p,hsa-miR-30e-3p,hsa-miR-30a-3p,hsa-miR-485-5p
circEPS 15	hsa_circ_000556 7	NM_005741	ZNF263	0.00052 950	hsa-miR-30d-3p,hsa-miR-30e-3p,hsa-miR-30a-3p,hsa-miR-192-3p
circEPS 15	hsa_circ_000556 7	NM_016480	PAIP2	0.00052 950	hsa-miR-620,hsa-miR-30d-3p,hsa-miR-30e-3p,hsa-miR-30a-3p
circEPS 15	hsa_circ_000556 7	NM_032315	SLC25A33	0.00055 855	hsa-miR-30d-3p,hsa-miR-30e-3p,hsa-miR-30a-3p
circEPS 15	hsa_circ_000556 7	NM_001429	EP300	0.00056 372	hsa-miR-138-5p,hsa-miR-30d-3p,hsa-miR-30e-3p,hsa-miR-30a-3p
circEPS 15	hsa_circ_000556 7	NM_145261	DNAJC19	0.00056 372	hsa-miR-30d-3p,hsa-miR-30e-3p,hsa-miR-30a-3p,hsa-miR-485-5p
circEPS 15	hsa_circ_000556 7	NM_033215	PPP1R3F	0.00059 950	hsa-miR-138-5p,hsa-miR-30d-3p,hsa-miR-30e-3p,hsa-miR-30a-3p
circEPS 15	hsa_circ_000556 7	NM_0012866 33	TRIM40	0.00059 950	hsa-miR-24-3p,hsa-miR-620,hsa-miR-627-5p,hsa-miR-485-5p
circEPS 15	hsa_circ_000556 7	NM_001414	EIF2B1	0.00063 687	hsa-miR-875-3p,hsa-miR-30d-3p,hsa-miR-30e-3p,hsa-miR-30a-3p
circEPS 15	hsa_circ_000556 7	NM_0011949 98	CEP152	0.00077 544	hsa-miR-30d-3p,hsa-miR-30e-3p,hsa-miR-30a-3p
circEPS 15	hsa_circ_000556 7	NM_138437	GPRASP2	0.00077 544	hsa-miR-30d-3p,hsa-miR-30e-3p,hsa-miR-30a-3p
circEPS 15	hsa_circ_000556 7	NM_003325	HIRA	0.00077 544	hsa-miR-30d-3p,hsa-miR-30e-3p,hsa-miR-30a-3p
circEPS 15	hsa_circ_000556 7	NM_0011998 18	ARMCX5-GPRASP2	0.00077 544	hsa-miR-30d-3p,hsa-miR-30e-3p,hsa-miR-30a-3p

circEPS15	hsa_circ_0005567	NM_002165	ID1	0.00077544	hsa-miR-30d-3p,hsa-miR-30e-3p,hsa-miR-30a-3p
circEPS15	hsa_circ_0005567	NM_153341	RNF19B	0.00094617	hsa-miR-30d-3p,hsa-miR-30e-3p,hsa-miR-30a-3p
circEPS15	hsa_circ_0005567	NM_001721	BMX	0.00103980	hsa-miR-30d-3p,hsa-miR-30e-3p,hsa-miR-30a-3p
circEPS15	hsa_circ_0005567	ENST00000391612	AL391152.1	0.00113913	hsa-miR-24-3p,hsa-miR-33a-5p,hsa-miR-33b-5p
circEPS15	hsa_circ_0005567	NM_145699	APOBEC3A	0.00124429	hsa-miR-30d-3p,hsa-miR-30e-3p,hsa-miR-30a-3p
circEPS15	hsa_circ_0005567	NM_001099921	MAGEB16	0.00124429	hsa-miR-875-3p,hsa-miR-492,hsa-miR-589-3p
circEPS15	hsa_circ_0005567	NM_152635	OIT3	0.00135540	hsa-miR-30d-3p,hsa-miR-30e-3p,hsa-miR-30a-3p
circEPS15	hsa_circ_0005567	NM_001190706	MTRNR2L9	0.00142453	hsa-miR-33a-5p,hsa-miR-33b-5p
circEPS15	hsa_circ_0005567	NM_001302998	LIPI	0.00142453	hsa-miR-33a-5p,hsa-miR-33b-5p
circEPS15	hsa_circ_0005567	NM_000329	RPE65	0.00147261	hsa-miR-30d-3p,hsa-miR-30e-3p,hsa-miR-30a-3p
circEPS15	hsa_circ_0005567	NM_025052	MAP3K19	0.00147261	hsa-miR-30d-3p,hsa-miR-30e-3p,hsa-miR-30a-3p
circEPS15	hsa_circ_0005567	NM_005926	MFAP1	0.00159603	hsa-miR-589-3p,hsa-miR-33a-5p,hsa-miR-33b-5p
circEPS15	hsa_circ_0005567	NM_000728	CALCB	0.00172578	hsa-miR-30e-3p,hsa-miR-30a-3p,hsa-miR-141-5p
circEPS15	hsa_circ_0005567	ENST00000594262	AC027307.3	0.00200478	hsa-miR-30d-3p,hsa-miR-30e-3p,hsa-miR-30a-3p
circEPS15	hsa_circ_0005567	NM_005114	HS3ST1	0.00200478	hsa-miR-30d-3p,hsa-miR-30e-3p,hsa-miR-30a-3p
circEPS15	hsa_circ_0005567	ENST00000366221	AL645728.1	0.00231052	hsa-miR-30d-3p,hsa-miR-30e-3p,hsa-miR-30a-3p
circEPS15	hsa_circ_0005567	NM_022054	KCNK13	0.00231052	hsa-miR-627-5p,hsa-miR-33a-5p,hsa-miR-33b-5p
circEPS15	hsa_circ_0005567	NM_152995	NFXL1	0.00231052	hsa-miR-24-3p,hsa-miR-30e-3p,hsa-miR-141-5p
circEPS15	hsa_circ_0005567	NM_012341	GTPBP4	0.00247371	hsa-miR-30d-3p,hsa-miR-30e-3p,hsa-miR-30a-3p
circEPS15	hsa_circ_0005567	NM_014500	HTATSF1	0.00258149	hsa-miR-33a-5p,hsa-miR-33b-5p
circEPS15	hsa_circ_0005567	NM_012458	TIMM13	0.00264391	hsa-miR-485-5p,hsa-miR-33a-5p,hsa-miR-33b-5p
circEPS15	hsa_circ_0005567	NM_005769	CHST4	0.00264391	hsa-miR-138-5p,hsa-miR-24-3p,hsa-miR-485-5p
circEPS15	hsa_circ_0005567	NM_199330	HOMER2	0.00264391	hsa-miR-30d-3p,hsa-miR-30e-3p,hsa-miR-30a-3p
circEPS15	hsa_circ_0005567	NM_004466	GPC5	0.00282124	hsa-miR-30d-3p,hsa-miR-30e-3p,hsa-miR-30a-3p

circEPS15	hsa_circ_0005567	NM_144574	WDR20	0.00282124	hsa-miR-145-3p,hsa-miR-33a-5p,hsa-miR-33b-5p
circEPS15	hsa_circ_0005567	NM_130386	COLEC12	0.00300579	hsa-miR-30d-3p,hsa-miR-30e-3p,hsa-miR-30a-3p
circEPS15	hsa_circ_0005567	NM_001010892	RSPH4A	0.00319767	hsa-miR-875-3p,hsa-miR-589-3p,hsa-miR-141-5p
circEPS15	hsa_circ_0005567	NM_005460	SNCAIP	0.00319767	hsa-miR-138-5p,hsa-miR-24-3p,hsa-miR-589-3p
circEPS15	hsa_circ_0005567	NM_006530	YEATS4	0.00319767	hsa-miR-30d-3p,hsa-miR-30e-3p,hsa-miR-30a-3p
circEPS15	hsa_circ_0005567	NM_213653	HFE2	0.00339697	hsa-miR-589-3p,hsa-miR-485-5p,hsa-miR-192-3p
circEPS15	hsa_circ_0005567	NM_024409	NPPC	0.00353189	hsa-miR-138-5p,hsa-miR-485-5p
circEPS15	hsa_circ_0005567	NM_173353	TPH2	0.00360379	hsa-miR-33a-5p,hsa-miR-33b-5p,hsa-miR-141-5p
circEPS15	hsa_circ_0005567	NM_004261	15-Sep	0.00360379	hsa-miR-24-3p,hsa-miR-33a-5p,hsa-miR-33b-5p
circEPS15	hsa_circ_0005567	NM_005817	PLIN3	0.00360379	hsa-miR-24-3p,hsa-miR-670-5p,hsa-miR-485-5p
circEPS15	hsa_circ_0005567	NM_176875	CCKBR	0.00381822	hsa-miR-30d-3p,hsa-miR-30e-3p,hsa-miR-30a-3p
circEPS15	hsa_circ_0005567	NM_015696	GPX7	0.00381822	hsa-miR-138-5p,hsa-miR-33a-5p,hsa-miR-33b-5p
circEPS15	hsa_circ_0005567	NM_001006938	TCEAL6	0.00381822	hsa-miR-30d-3p,hsa-miR-30e-3p,hsa-miR-30a-3p
circEPS15	hsa_circ_0005567	NM_003031	SIAH1	0.00381822	hsa-miR-30d-3p,hsa-miR-30e-3p,hsa-miR-30a-3p
circEPS15	hsa_circ_0005567	NM_001114938	CCDC17	0.00405954	hsa-miR-875-3p,hsa-miR-589-3p
circEPS15	hsa_circ_0005567	NM_003960	NAT8	0.00462157	hsa-miR-33a-5p,hsa-miR-33b-5p
circEPS15	hsa_circ_0005567	NM_005391	PDK3	0.00462157	hsa-miR-30e-3p,hsa-miR-30a-3p
circEPS15	hsa_circ_0005567	NM_003142	SSB	0.00521757	hsa-miR-589-3p,hsa-miR-141-5p
circEPS15	hsa_circ_0005567	NM_153707	SAXO1	0.00584713	hsa-miR-670-5p,hsa-miR-492
circEPS15	hsa_circ_0005567	NM_003154	STATH	0.00584713	hsa-miR-33a-5p,hsa-miR-33b-5p
circEPS15	hsa_circ_0005567	NM_006346	PIBF1	0.00584713	hsa-miR-33a-5p,hsa-miR-33b-5p
circEPS15	hsa_circ_0005567	NM_006794	GPR75	0.00650983	hsa-miR-30e-3p,hsa-miR-30a-3p
circEPS15	hsa_circ_0005567	NM_012130	CLDN14	0.00650983	hsa-miR-30e-3p,hsa-miR-30a-3p
circEPS15	hsa_circ_0005567	NM_016552	ANKMY1	0.00650983	hsa-miR-24-3p,hsa-miR-670-5p

circEPS15	hsa_circ_0005567	NM_002282	KRT83	0.00650983	hsa-miR-138-5p,hsa-miR-589-3p
circEPS15	hsa_circ_0005567	NM_030577	TMEM177	0.00650983	hsa-miR-24-3p,hsa-miR-670-5p
circEPS15	hsa_circ_0005567	NM_080742	B3GAT2	0.00650983	hsa-miR-589-3p,hsa-miR-141-5p
circEPS15	hsa_circ_0005567	NM_022147	RTP4	0.00720526	hsa-miR-30e-3p,hsa-miR-30a-3p
circEPS15	hsa_circ_0005567	NM_001279	CIDEA	0.00720526	hsa-miR-24-3p,hsa-miR-589-3p
circEPS15	hsa_circ_0005567	NM_017864	INTS8	0.00720526	hsa-miR-30d-3p,hsa-miR-30a-3p
circEPS15	hsa_circ_0005567	NM_005097	LGI1	0.00793301	hsa-miR-33a-5p,hsa-miR-33b-5p
circEPS15	hsa_circ_0005567	NM_003770	KRT37	0.00869269	hsa-miR-620,hsa-miR-627-5p
circEPS15	hsa_circ_0005567	NM_000273	GPR143	0.00869269	hsa-miR-138-5p,hsa-miR-627-5p
circEPS15	hsa_circ_0005567	NM_018146	RNMTL1	0.00869269	hsa-miR-138-5p,hsa-miR-492
circEPS15	hsa_circ_0005567	NM_001017964	YDJC	0.00869269	hsa-miR-33a-5p,hsa-miR-33b-5p
circEPS15	hsa_circ_0005567	ENST00000313548	CHDC2	0.00948388	hsa-miR-138-5p,hsa-miR-24-3p
circEPS15	hsa_circ_0005567	NM_022838	ARMCX5	0.00948388	hsa-miR-33a-5p,hsa-miR-33b-5p
circEPS15	hsa_circ_0005567	NM_031965	GSG2	0.00948388	hsa-miR-30d-3p,hsa-miR-589-3p
circEPS15	hsa_circ_0005567	ENST00000439873	AL354993.1	0.01030620	hsa-miR-33a-5p,hsa-miR-33b-5p
circEPS15	hsa_circ_0005567	NM_025165	ELL3	0.01115925	hsa-miR-30e-3p,hsa-miR-30a-3p
circEPS15	hsa_circ_0005567	NM_001205271	PRSS46	0.01115925	hsa-miR-138-5p,hsa-miR-24-3p
circEPS15	hsa_circ_0005567	NM_007098	CLTCL1	0.01115925	hsa-miR-24-3p,hsa-miR-485-5p
circEPS15	hsa_circ_0005567	NM_016568	RXFP3	0.01204265	hsa-miR-138-5p,hsa-miR-192-3p
circEPS15	hsa_circ_0005567	NM_152760	SNX32	0.01295599	hsa-miR-145-3p,hsa-miR-670-5p
circEPS15	hsa_circ_0005567	NM_002252	KCNS3	0.01295599	hsa-miR-589-3p,hsa-miR-33b-5p
circEPS15	hsa_circ_0005567	NM_002982	CCL2	0.01295599	hsa-miR-33a-5p,hsa-miR-33b-5p
circEPS15	hsa_circ_0005567	NM_004882	CIR1	0.01295599	hsa-miR-620,hsa-miR-589-3p
circEPS15	hsa_circ_0005567	NM_004390	CTSH	0.01389891	hsa-miR-138-5p,hsa-miR-627-5p

circEPS 15	hsa_circ_000556 7	NM_005514	HLA-B	0.01487 102	hsa-miR-30e-3p,hsa-miR-30a-3p
circEPS 15	hsa_circ_000556 7	NM_001382	DPAGT1	0.01487 102	hsa-miR-589-3p,hsa-miR-485-5p
circEPS 15	hsa_circ_000556 7	NM_030960	SPACA1	0.01587 194	hsa-miR-875-3p,hsa-miR-670-5p
circEPS 15	hsa_circ_000556 7	NM_005499	UBA2	0.01587 194	hsa-miR-33a-5p,hsa-miR-33b-5p
circEPS 15	hsa_circ_000556 7	NM_003789	TRADD	0.01587 194	hsa-miR-145-3p,hsa-miR-485-5p
circEPS 15	hsa_circ_000556 7	NM_000576	IL1B	0.01587 194	hsa-miR-30e-3p,hsa-miR-30a-3p
circEPS 15	hsa_circ_000556 7	NM_003757	EIF3I	0.01587 194	hsa-miR-24-3p,hsa-miR-620
circEPS 15	hsa_circ_000556 7	NM_003126	SPTA1	0.01690 131	hsa-miR-589-3p,hsa-miR-141-5p
circEPS 15	hsa_circ_000556 7	NM_000756	CRH	0.01690 131	hsa-miR-24-3p,hsa-miR-875-3p
circEPS 15	hsa_circ_000556 7	NM_000779	CYP4B1	0.01690 131	hsa-miR-627-5p,hsa-miR-192-3p
circEPS 15	hsa_circ_000556 7	NM_024783	AGBL2	0.01795 874	hsa-miR-33a-5p,hsa-miR-33b-5p
circEPS 15	hsa_circ_000556 7	NM_031950	FGFBP2	0.01795 874	hsa-miR-24-3p,hsa-miR-875-3p
circEPS 15	hsa_circ_000556 7	NM_0012584 28	SLC4A9	0.01904 388	hsa-miR-24-3p,hsa-miR-670-5p
circEPS 15	hsa_circ_000556 7	NM_020820	PREX1	0.01904 388	hsa-miR-138-5p,hsa-miR-670-5p
